# Bacterial diversity of stingless bee honey in Yunnan, China: isolation and genome sequencing of a novel acid-resistant *Lactobacillus pentosus* (*SYBC-MI*) with probiotic and L. tryptophan producing potential via millet fermentation

**DOI:** 10.3389/fbioe.2023.1272308

**Published:** 2023-12-01

**Authors:** Samra Basharat, Tiantian Meng, Lixin Zhai, Asif Hussain, Sahibzada Muhammad Aqeel, Salman Khan, Obaid Ullah Shah, Xiangru Liao

**Affiliations:** ^1^ Key Laboratory of Industrial Biotechnology, Ministry of Education, Jiangnan University, Wuxi, China; ^2^ Henan Key Laboratory of Biomarker Based Rapid-detection Technology for Food Safety, Institute of Molecular Detection Technology and Equipment, Xuchang University, Xuchang, Henan, China; ^3^ National Engineering Research Center for Cereal Fermentation and Food Biomanufacturing, Jiangnan University, Wuxi, China; ^4^ Collaborative Innovation Center of Nanfan and High-Efficiency Tropical Agriculture, School of Tropical Crops, Hainan University, Haikou, China

**Keywords:** bacterial diversity, stingless bee honey, acid resistant lactic acid bacteria, genome analysis, millet fermentation, L. tryptophan, probiotic potential

## Abstract

Stingless bee (*Hymenoptera, Apidae*, and *Trigona*) honey is a remarkable “miracle liquid” with a wide range of medical benefits for conditions including gastroenteritis, cataracts, and wound healing. Our study aimed to isolate, identify, and characterize acid-resistant *Lactobacillus* spp. from sour honey distributed in Yunnan, China. To assess the safety of an entirely novel *Lactobacillus pentosus* strain, S4 (OM618128), based on probiotic property evaluation and whole-genome sequencing analysis. A 16S rRNA gene high-throughput sequencing analysis showed that *Lactobacillus* was abundant at the genus level in sour honey. Seven *Lactobacillus* strains (viz. S1–7) were isolated from sour honey using a multiple-anaerobic culture enrichment method. One potential acid-resistant isolate, *Lactobacillus* sp. S4, was obtained after screening the seven *Lactobacillus* isolates, and it had the highest lactic acid production (17.62 g/L), followed by *Lactobacillus* sp. S3 (17.07 g/L). Phylogenetic and comparative analyses of conserved sequence regions have shown that all seven strains are phylogenetically located in the *Lactobacillus pentosus* sub-cluster. In *L. pentosus SYBC-MI*, there is a circular chromosome (3288615 bps) and 11,466 bps plasmids. GC content is 44.03%. The number of predicted genes is 3,129, with 16 rRNAs and 74 tRNAs present. During the fermentation of foxtail millet by seven *Lactobacillus pentosus* (S1–7) strains isolated from sour honey, a potential tryptophan accumulating isolate, *Lactobacillus pentosus* S4, was obtained, which could reach a maximum tryptophan content of 238.43 mgL^-1^ that is 1.80 times the initial tryptophan content in the fermentation broth. This strain has strong acid tolerance, salt tolerance, and fermentation acid production abilities. This strain degrades nitrite at a rate of over 99%, and it has high probiotic potential as well. This project has established a solid foundation for further exploring the excellent lactic acid bacteria in sour honey. It is also investigating the key taxa and their role in the environment. According to the results of our studies, these LAB isolates provide a lot of potential for use in the future, as a source of probiotics for human, animals, and starter cultures for food applications.

## Highlights

➢ Stingless bee honey (SBH) has excellent medicinal value for human health and may contain acid-resistant lactic acid bacteria. Our work is the first report highlighting the presence of *Lactobacillus pentosus* in the sour honey of stingless bees from Yunnan, China.
➢ This knowledge improves the understanding of community structure in SBH bacteria at the genus level. In addition, *Lactobacillus* strains with strong acid-resistant levels and acid-producing capacities were isolated from SBH, and they have potential applications in the food and feed industries.
➢ The isolation of *lactobacillus* with probiotic potential and the evaluation of L-tryptophan accumulation can be a fascinating research area with potential applications in the development of novel probiotic products with enhanced health benefits. Fermentation of millet using lactic acid bacteria has a long history, and millet itself has substantial value for functional health foods. Probiotic fermentation adds a new flavor and nutritional value to traditional grains.

## 1 Introduction

Stingless bees are widely distributed in tropical and subtropical areas of the world, with over 500 known species, most of which are found in Latin America, the Australian continent, Africa, and East and South Asia (Vit et al., 1994; Abd Jalil et al., 2017). Their honey has excellent medicinal value related to improving human health (Irish et al., 2008; Braghini et al., 2022). The tribe Meliponini, also known as stingless bees, is the biggest group of eusocial bees on Earth, with more than 500 species, which is 50–60 times more than its competing counterparts, the honeybees (Apis sp.; Giraffa et al., 2010; Sadiq et al., 2019). They have been on earth for 66 million years, longer than Apis species. Their honey is antimicrobially more active and lower in sugar than that of most honeybees (Rosli et al., 2020; Menezes et al., 2013; Baharudin et al., 2021). However, compared to honey bees, stingless bees have received relatively less research (Leroy and De Vuyst, 2004). The stingless wasp (Trigona), also known as the “acid wasp” in China, is mainly found in Xishuangbanna, Yunnan, and Hainan. It is a close relative of the genus Mellipona, found in Brazil and Mexico (Olofsson et al., 2014). Stingless bees are smaller than other honeybees, so they are more flexible and can penetrate most plants to collect pollen, making them an ideal resource for insect pollination (Guo et al., 2011; Tannock, 2004).

Stingless bee honey, also known as sour honey because of its low pH and pronounced acidity, is a natural honey consumed locally in Yunnan (Audisio et al., 2011; Audisio, 2017). Honeybees are natural microbial collectors who spread microorganisms through nectar collection and pollination. Stingless bees being one of them produce sour honey that may be an effective source of novel lactic acid bacteria. Stingless bee honey has a sweet-to-sour taste, low viscosity, and slow crystallization (Tajabadi, Mardan, Manap, et al., 2013). Unlike normal honey, sour honey has a high level of acidity and moisture but lower sugar content, amylase activity, and 5-hydroxymethylfurfural (5-HMF; Wang et al., 2018), making it a natural honey long consumed by local people in Yunnan. Miraculously, in the pristine rainforests of Xishuangbanna, native bees often give up wild plants with high nutritional value to Yunnan acid bees to harvest (e.g., wild yellow lily, wild panax, and ginseng). Studies have shown that acid honey has excellent medicinal value for human health (Vit et al., 1994; Floch and Hong-Curtiss, 2002; Pieniz et al., 2014), and its excellent probiotic capacity depends to some extent on its antibacterial ability (Vit et al., 1994; Rosli et al., 2020). In 2013, Mercês et al. found that sour honey was more effective than sugar solutions in inhibiting bacterial growth (Mercês et al., 2013). In 2016, Kek et al. showed that sour honey has strong antioxidant properties and a safe and effective chemopreventive effect against colon cancer (Kek et al., 2017). In addition, acid honey can be ingested directly or used as a food additive because it has been demonstrated to be useful against conditions like chemically induced cataracts, gastroenteritis, and wound healing (Dong et al., 2020; Hamzah et al., 2020).

According to recent findings, stingless bee honey contains a wide variety of microorganisms, including eight bacterial species, 71 families, 155 genera, and 70–75 species with antibiotic effects against pathogenic bacteria (Laslo et al., 2020). Actinobacteria and Firmicutes are the most prevalent groups of bacteria in bee digestive tracts, and their microbiota evolve through eating plant material such as nectar and pollen. Lactic acid bacteria (LAB) from several taxa have been associated with insect hosts (Hurtado et al., 2011; Morais et al., 2012). The category of Gram-positive bacteria that do not produce budding spores and mainly produce lactic acid during sugar fermentation may indicate that there are acid-resistant LAB strains in the honey. However limited studies have been conducted to isolate and identify acid-resistant LAB from sour honey (Tuksitha et al., 2018; Rosli et al., 2020). The majority of recent microbiota research has focused on bees, determining microbial diversity and abundance using metagenomics and culture-dependent approaches (cultivation of bacteria; Giraffa et al., 2010; Poornachandra Rao et al., 2015). Most LAB are non-pathogenic and awarded “generally recognized as safe” (GRAS) status (Sadiq et al., 2019), widely used in all aspects of the food fermentation industry. Due to their positive effects on their host, such as enhancing disease resistance and improving the reproductive system, some LAB are known as probiotics (Tannock, 2004). With an increased understanding of the nutritional values and functional activities of LAB and the rapid development of biotechnology, probiotic LAB strains are widely used in the food fermentation industry (Leroy and De Vuyst, 2004), the feed industry, and biomedical fields (Guo et al., 2011). Screening functionally active LAB is important in the development of functional foods, as the lactic acid they produce helps prevent spoilage and the growth of pathogenic microorganisms during the production and storage of fermented foods (Olofsson et al., 2014; Petrova et al., 2015; Kwong and Moran, 2016; Behera et al., 2018; Torres-Moreno et al., 2021).

*Lactobacillus pentosus* is a facultative heterofermentative lactic acid bacterium (LAB) with one of the biggest genomes known among LABs. It can obtain energy from a variety of carbohydrates and has excellent adaptation to a wide range of niches, including dairy products, vegetables, wine, and both people’s and animals’ gastrointestinal tracts. A unique acid-resistant *Lactobacillus pentosus* strain has been isolated and found in Yunnan, China, where the bacterial diversity of stingless bee honey has been examined, which may contribute to its distinct flavor and therapeutic effects. Previous studies have demonstrated that the medicinal benefits of stingless bee honey are mostly associated with the production of bacteriocins and organic acids from the metabolism of the microorganisms in it (Ngalimat et al., 2019)**.** By preventing the growth of dangerous bacteria, reducing nitrite levels, and improving the health of the gut microbiota *in vitro*, *lactobacillus* strains can improve food quality and be used as potential probiotics (Audisio et al., 2011; Thomas and Kharnaior, 2021). LABs must be acid-resistant to be used as probiotics because the stomach pH, 2.0 to 3.0, takes 2–4 h to pass through (Tajabadi, Mardan, Saari, et al., 2013; Shao et al., 2021; Baharudin et al., 2021). Lactic acid bacteria come from a wide range of sources, including fermented foods (Behera et al., 2018)**,** the human body, and animals (Petrova et al., 2015).

In China, traditional millet fermented foods such as millet bakery and millet steamed buns have a long history. The popularity of millet-fermented beverages in China has risen in recent years due to millet vinegar, the earliest wine made from it millet directly has many benefits (Osman, 2011). After fermentation, carbohydrates, proteins, fats, phytic acid, fiber, and other large molecules are broken down into smaller molecules, which are more beneficial to human absorption and utilization. Millet is rich in eight essential amino acids: threonine, leucine, Isoleucine, histidine, lysine, methionine, tryptophan, and valine. The content of most of these amino acids in millet is higher than in bulk grains such as wheat, rice, and corn, especially methionine and tryptophan. (Hu et al., 2012; Wang et al., 2018).

Fermented millet can be a beneficial food supplement, especially now that urban dwellers consume refined rice as their main diet. Fermented millet can be cooked into porridge, added with salt, sugar, and/or skim milk for direct use, or mixed and processed with other grains and steamed products (Irish et al., 2008; Mercês et al., 2013; Syed Yaacob et al., 2018; Wong et al., 2019). Studies have shown that millet fermentation results in improved protein extractability, especially the albumin and globulin fractions. This indicates the increased availability of proteins in fermented millet. These changes may be the result of tannin and phytic acid breakdown and increased microbial protease activity (Banwo et al., 2021). In recent years, the production of active millet peptides and phenolic acids with antibacterial and antioxidant properties using fermented millet has been used in medicine.

Furthermore, in China, stingless bee honey is widely used by the local people of Yunnan, but it has not been studied as a potential source of novel acid-resistant lactic acid bacteria. Research into the bacterial diversity of stingless bee honey and the isolation of novel bacterial strains may provide valuable insights into the possible health benefits of honey consumption and the use of these bacteria in various industrial applications. We collected honey samples from different sources to assess the bacterial diversity of stingless bee honey in Yunnan. The isolation and identification of the novel acid-resistant *lactobacillus* strain likely involved screening honey samples for *lactobacillus* species, then testing their ability to survive under acidic conditions (which is important for their potential use in fermentation processes). The strains have been further characterized through biochemical tests, DNA sequencing, and other methods to determine its unique properties and potential applications. This study aimed to isolate, identify, and characterize acid-resistant *Lactobacillus* spp. from sour honey distributed in Yunnan, China. A high-throughput sequencing analysis was used to describe bacterial communities in sour honey from four different habitats in Yunnan. Several *Lactobacillus* spp. strains were isolated using a multi-round anaerobic bacterial culture enrichment method. Whole genome sequence analysis, acid-producing capacity, probiotic activity and tryptophan accumulation ability of a novel acid-resistant *Lactobacillus* spp. isolated from stingless bee honey (sour honey) are described.

## 2 Materials and methods

### 2.1 Sampling of sour honey

Four types of stingless bee honey samples were collected from two professional beekeepers on three separate occasions during June 2020, with four biological replicates per habitat. The study area is located at tropical Xishuangbanna forests (53°17′–53°30′ N, 122°06′–122°27′ E) in Yunnan Province, China. Each sample was aseptically extracted with a 25-ml sterile syringe and stored in a 50 ml sterile glass-sealed container after sample collection. These samples were immediately frozen and transported immediately to Jiangnan University’s laboratory in Wuxi, China, for further processing. The fresh sour honey samples were numbered Y1–4 and stored in a refrigerator at 4°C for short-term use.

### 2.2 Analysis of bacterial diversity

Using Xishuangbanna native sour honey as experimental material, the sour honey samples were domesticated and cultured with MRS medium for five rounds. The bacterial 16S rRNA gene were sequenced using high-throughput sequencing technology for domesticated sour honey samples to compare and analyze the composition of microbial communities in the sour honey samples and observe the domestication.

#### 2.2.1 DNA extraction and PCR amplification

A NucleoSpin^®^96 Soil kit was used to extract total bacterial DNA from each sample, and the samples were stored in a refrigerator at −20°C for further processing. The DNA samples from honey were mixed to increase the DNA concentration and to make the samples more representative. After centrifuging for 10 min at 5,000 g, all separated cells were extracted and placed in a microcentrifuge tube. The bacteria pellet was resuspended in 180 mL of enzymatic lysis buffer (20 mM Tris-Cl’, pH = 8.0; 2 mM sodium salt of ethylene diamine tetraacetic acid (EDTA); 1.2% Triton X-100 solution; and 20 mg/mL lysozyme, QIAGene, Hilden, Germany) and incubated for at least 30 min at 37°C. A tube was filled with 200 mL of buffer AL (QIAGene, Hilden, Germany) and 25 L of proteinase K. The mixture was vortexed and incubated at 56°C for 30 min. The sample was added to a volume of 200 L of ethanol (96%; HmBg Chemicals, Germany) and carefully mixed using a vortex. The sample was loaded into a 2 mL collection tube and spun at 6,000 g for 1 min on a DNeasy Mini spin column (QIAGene, Hilden, Germany). After inserting the DNeasy Mini spin column into a fresh 2 mL collection tube and adding 500 L of buffer AW1, the mixture was centrifuged at 6000g for 1 min. After that, the buffer AW2 (QIAGene, Hilden, Germany) was added to a fresh 2 mL collection tube, and the mixture was once more centrifuged for 3 min at 20,000 g. After that, 200 L of buffer AE were immediately transferred to the DNeasy membrane by placing the DNeasy Mini spin column in a clean 2-mL micro centrifuge tube. DNA samples were extracted in 150 L of double-distilled water and kept at - 24°C in a freezer. DNA purity was measured using a spectrophotometer and the ratios of absorbance at 260 and 280 nm.

PCR was performed using common primers 338F (5′-ACT​CCT​ACG​GGA​GGC​AGC​A-3′) and 806R (5′-GGACTACHVGGGTWTCTAAT-3′) for the V3–V4 region of the bacterial 16S rRNA gene (Wu et al., 2013). The PCR reagents (Table 1)**,** and the amplification conditions were 25 μL reaction volume, including 0.2 μL 5 U/μL Taq DNA polymerase, 2 μL dNTPs, 2.5 μL MgCl_2_, reaction buffer (10× Taq Buffer (Mg2+), 5 μL forward primer (27F), 5 μL reverse primer (1429R), 8.3 μL nuclease-free water, and 2 μL of approximately 10–100 ng/μL genomic DNA. The following were the temperature cycling conditions: 10 min of initial heating at 94°C, followed by 30 cycles of denaturation for 30 s at 94°C, annealing for 30 s at 58°C, and extension for 45 s at 72°C, and finally a final incubation of 5 min at 72°C.

**TABLE 1:** PCR Reagents and reaction conditions.

*Component*	*Volume of one reaction* (µl)
PCR product of previous step	2.5
2×KAPA HiFi Hot Start Ready Mix	12.5
Specific Forward Primer (25 µM)	0.25
Specific Reverse Primer (25 µM)	0.25
PCR Grade Water	9.5
Total volume	25

#### 2.2.2 Illumina HiSeq high-throughput sequencing

High-throughput sequencing was performed by using an Illumina HiSeq 2,500 platform (PE250). HiSeq database construction and gene sequencing tasks were performed by Biomarker Technologies Co., Ltd. (Beijing, China). DNA sequencing, splicing, quality control, advanced bioinformatics analysis, and sequence data analysis were done with assistance from this company. Library construction and sequencing was done by extracting the total DNA of the samples. Primers were designed according to the conserved region, sequencing connector is added at the end of the primers, PCR amplification is carried out, and the products are purified, quantified and homogenized to form a sequencing library. The constructed library is subjected to the quality control of library first, and Illumina HiSeq 2,500 sequences the library that passes the quality control. The raw image data files obtained from high-throughput sequencing (e.g., Illumina HiSeq and other sequencing platforms) were analyzed by base calling to be converted into raw sequenced reads, and the results were stored in the FASTQ file format, which contains the sequence information of the sequenced, reads as well as their corresponding sequencing quality information.

Information analysis data preprocessing involves the following three steps: 1) Raw reads filtering: First, Trimmomatic v0.33 software was used to filter the Raw Reads obtained from sequencing; and then use cutadapt 1.9.1 software to identify and remove primer sequences, to obtain high-quality Reads that do not contain primer sequences. 2) High-quality Reads splicing: FLASH v1.2.7 software was used to splice the high quality reads of each sample by overlap, and the spliced sequences obtained were Clean Reads. 3) Remove chimeras: UCHIME v4.2 software used to identify and remove chimeric sequences, and obtain the final effective data (Effective Reads).

Data quality was assessed by counting the number of sequences of the samples at each stage of data processing. The data were mainly evaluated by counting the number of sequences at each stage, sequence length and other parameters. The species diversity of individual samples was examined using an alpha-diversity analysis, and each sample’s Chao1, Shannon, and Simpson indices at the 97% similarity level were calculated (Jiang et al., 2020; Suphaphimol et al., 2020). The bacterial community structure of sour honey was further analyzed using the quality information of tested samples and the obtained microbial diversity-related information. SILVA was used as the reference database to annotate the feature sequences taxonomically using a simple Bayesian classifier, the taxonomic information corresponding to each feature could be obtained. We then calculated the community composition of each sample at each level (phylum, class, order, family, genus, and species), generated the abundance table of species at different taxonomic levels by using QIIME software, and then plotted the community structure of the samples at each taxonomic level by using R tools. The community structure of each sample was then plotted using the R language tool. The original feature list may contain very low abundance features (species abundance less than 0.005%), the low abundance features were filtered to get the final feature list and counted the number of annotated-to-species tags for each level in each sample.

### 2.3 Isolation and identification of novel acid resistant strain “S4”

The sour honey samples were cultured in de Man, Rogosa, and Sharpe (MRS) liquid medium through five rounds with anaerobic enrichment (cultured at 37°C for 48 h), and the resulting bacterial solution was diluted with 0.9% sterile normal saline. Then, 100 μL aliquot of each dilution was spread onto an MRS agar separation medium containing 2% CaCO_3_. The plates were incubated anaerobically at 37°C for 48 h. Colonies (acid-producing bacteria) with a transparent circle around them were selected and streaked on MRS agar medium (Syed Yaacob et al., 2018). The region in which the culture medium around the colony changed from cloudy to clear was the calcium-soluble circle, indicating that CaCO_3_ was hydrolyzed due to acid production by the strain. This selection process was repeated four times. Pure bacterial isolates were preserved in nutritional broth with 20% (v/v) glycerol at −80°C.

### 2.4 Physiological and biochemical characterization

The LAB strains were firstly tested for their biochemical and physiological properties. The morphological characteristics were determined using gram staining, in which gram-positive LAB strains appear purple. The preliminarily screened LAB strains were streaked on MRS broth and cultured upside down at 37°C for 48 h to observe the colors, shapes, sizes, and other characteristics of the colonies All isolates were tested for viability at different temperatures (15, 30, 35, and 45°C) salinities (6, 8, and 10% NaCl concentrations) and spore forming test. Typical spore count tests require heating a reconstituted powder sample to 80°C for 12 min before cooling, cultivating, and counting colonies.

The isolates were also tested for catalase test, H_2_S production, gelatin liquefaction, indole production, bile salt hydrolase, and starch hydrolysis (Poornachandra Rao et al., 2015)**.**

#### 2.4.1 Carbohydrate fermentation acid production test

In this study, L-arabinose, D-cellulose-disaccharide, D-galactose, D-lactose, D-maltose, D-mannose, D-triose, L-rhamnose, D-ribose, D-sucrose, D-xylose, L-xylose, D-fructose, D-sorbitol, L-sorbose, inositol, and glycerol were added to the poly peptone yeast extract (PY) basal medium, independently, and then, they were used to characterize and assess the LAB isolates’ ability to utilize various carbohydrate-family substrates. Generally, 0.5%–1% (w/v) of the carbohydrates concentration is used for carbohydrate fermentation in 1L of basal medium.

Next, each isolate was added at a rate of 1%, and each strain was incubated for 75 min at a temperature between 30°C and 37°C. 96-well microplates were used for the test (Jiang et al., 2020). It was considered positive proof of carbohydrate fermentation when the color of the media changed from purple to yellow. Furthermore, the glucose fermentation test assessed acid and gas production to distinguish the LAB fermentation type, homo-fermentative or hetero-fermentative. A soft agar column was prepared by adding 3% glucose, 0.05% Tween-80, and 0.6% agar to the PY basal medium. To facilitate the observation of acid production, 1.4 mL bromothymol blue-methyl red reagent at a concentration of 1.6 g/100 mL was added to each test tube as an indicator. Then, many fresh and highly active strains were inoculated and cultured at 37°C for 48 h. The change in the color of the bromothymol blue-methyl red reagent indicates the degree of acid production.

#### 2.4.2 Acid production from glucose fermentation test

The acid production from glucose fermentation gas production test aims to distinguish the type of fermentation of lactic acid bacteria, whether it is homo- or hetero-lactic fermentation. A pure culture’s inoculum is aseptically transferred to a sterile tube containing bromothymol blue-methyl red as an indicator. Results are obtained after a 24-h incubation period at 35–37 C using the infected tube. The change in the color of the bromothymol blue-methyl red reagent indicates the degree of acid production (As described in section 2.4.1). For details, refer to the work Taxonomic identification and experimental methods of lactic acid bacteria (Jarocki et al., 2020).

#### 2.4.3 Cold field emission scanning electron microscopy observation

The surface micromorphology of *Lactobacillus* strain S4 was observed using a Hitachi SU8220 cold field emission scanning electron microscope (FESEM; JEOL, Japan). An area of around 3 × a mm was mounted on an SEM metal mount, and gold nanoparticles were sputtered onto the sample surface for 2 minutes. Then, using an Everhart-Thornley Detector and a high vacuum, the sputtered samples were scanned with a QuantaTM Field emission gun high-resolution scanning electron microscope at 3.0 kV (Abol-Fotouh et al., 2020).

#### 2.4.4 Molecular identification

A 16S rRNA sequencing were performed for the molecular identification. A pair of bacterial universal primers, designated 27F (5′-AGRGTTTGATYNTGGCTCAG-3′) and 1492R (5′-TASGGHTACCTTGTTASGACTT-3'; Abol-Fotouh et al., 2020) were designed by Tianlin Biotechnology Co. (Shanghai, China) and were used to amplify the conserved portions of bacterial 16S rRNA genes. The 16S rRNA sequences of the sequenced strains were compared with the homology of the reported strains in the NCBI microbial identification system (https://www.ncbi.nlm.nih.gov/) using the BLASTn algorithm to derive the genus information of the strains. The results showed that all seven isolates had the highest homology with *Lactobacillus pentosus* with 99.47%, 99.79%, 99.89%, 99.26%, 99.47%, 99.47%, 99.47% and 99.37% similarity, respectively. The sequences from each isolate were compared with sequences in the NCBI Reference Sequence Database for taxonomic identification using the advanced BLAST similarity search option (available at https://blast.ncbi.nlm.nih.gov/Blast.cgi; Suphaphimol et al., 2020). The nucleotide sequences of bacterial strains in this study (S1-S7) have been submitted to Gene Bank under following accession numbers OM618122, OM618126, OM618127, OM618128, OM618129, OM618130, OM618135 respectively on the link mention below;

Lactiplantibacillus pentosus strain S1 16S ribosomal RNA gene, partial - Nucleotide - NCBI (nih.gov).

Lactiplantibacillus pentosus strain S2 16S ribosomal RNA gene, partial - Nucleotide - NCBI (nih.gov).

Lactiplantibacillus pentosus strain S3 16S ribosomal RNA gene, partial - Nucleotide - NCBI (nih.gov).

Lactiplantibacillus pentosus strain S4 16S ribosomal RNA gene, partial - Nucleotide - NCBI (nih.gov).

Lactiplantibacillus pentosus strain S5 16S ribosomal RNA gene, partial - Nucleotide - NCBI (nih.gov).

Lactiplantibacillus pentosus strain S6 16S ribosomal RNA gene, partial - Nucleotide - NCBI (nih.gov).

Lactiplantibacillus pentosus strain S7 16S ribosomal RNA gene, partial - Nucleotide - NCBI (nih.gov).

Ten model strains with high 16S rRNA gene sequence homology with seven acid honey isolates within the genus *Lactobacillus* were then retrieved from the GenBank (https://www.ncbi.nlm.nih.gov/) and EzTaxon (http://www.ezbiocloud.net/) databases. A phylogenetic tree was constructed by employing the computer software MEGA X (available at https://www.megasoftware.net/) with the neighbor-joining method using the 16S rRNA gene sequence analysis (Kumar and Kumar, 2015).

### 2.5 Whole-genome sequence analysis of strain S4

#### 2.5.1 Genome sequence assembly and analysis

The genome of strain S4 was sequenced using the Illumina NovaSeq/Oxford Nanopore ONT platform. The required, genomic libraries were constructed using the Illumina TruSeq DNA Sample Preparation Guide (standard Illumina TruSeq Nano DNA LT library), and total DNA was detected using a fluorescent dye (Quant-iT PicoGreen dsDNA Assay Kit). The standard Sanger variant calculation and the early Solexa pipeline (e.g., Illumina Genome Analyzer software) were used to calculate the quality value or Q value, i.e., the result of a rounded mapping of the base read error rate ‘P’. Third-generation single-molecule sequencing data assembly for the Genomic sequence assembly: the 3-generation downstream data were assembled using HGAP (Hierarchical genome-assembly process; Chin et al., 2016) and CANU (Celera Assembler designed for high-noise single-molecule sequencing) (Sergey et al., 2017) software.

#### 2.5.2 Gene prediction and annotation

Gene prediction and annotation were achieved using NCBI prokaryotic Genome Annotation Pipeline. GeneMarkS software (V4.17) was used for protein-coding gene prediction in bacterial genomes. RepeatMasker (Versionopen-4.0.5) software was used to predict repetitive sequences, as well as TRF (Tandem Repeats Finder, V4.07b), ribosomal RNA (rRNA) prediction by RNAmmer software (V1.2), transfer RNA (tRNA) prediction by tRNAscan-SE software (V1.3.1), and small RNA (sRNA) determination by the program “cmsearch” (V1.1rc4). The tRNAscan-SE software is used to predict tRNAs’ tRNA region and secondary structure. PhiSpy was used to predict the presence of prophages in the genome (Kant et al., 2011). CAZy (Carbohydrate-Active enzymes Database) was used to check the carbohydrate-active enzymes (Anderson and Ricigliano, 2017). This database mainly contains enzymes related to glycosidic bonds.

Using the databases for the Kyoto Encyclopaedia of Genes and Genomes (KEGG), Gene Ontology (GO), and Clusters of Orthologous Groups (COG), functional annotation of predicted protein-coding sequences of acid resistance *Lactobacillus sp.* was carried out. The CRISPR finder was used to predict DRs (forward repeats) and spacers (spacer regions) in the whole genome (Mazhar et al., 2020). GO annotation of protein-coding genes was done using BLAST2GO software (Hurtado et al., 2011; Cochrane and Vederas, 2016), and GO annotation was done using the default parameters of BLAST2GO. Software called Circos was used to build genome visualization, and the antibiotic Resistance Genes Databases Blast Server was used to search for antibiotic resistance (Rosli et al., 2020).

### 2.6 Analysis of the probiotic potential of isolated strain

The isolates were examined for different probiotic qualities after identification up to the strain level. Probiotics were evaluated for acid resistance at pH 2, 3, and 4 compared to pH 7 because the pH of the human stomach is 2.

#### 2.6.1 Acid and bile tolerance assay

The strains were cultured in MRS broth for 24 h at 37°C. Bacterial cells in MRS broth were adjusted to a range of pH values (2.0–4.0) with 1.0 M HCl to evaluate the isolated strains’ resistance to low pH and bile salts. The suspension of strains (2% v/v) was inoculated into unadjusted MRS broth (as a control; pH 7.0) and cultivated at 37°C for 4 h. MRS broth was supplemented with bile salts (oxgall) concentrations of 0.2, 0.3, 0.4, 0.5, and 1.0% (w/v). Plating on MRS plates after a 10-fold dilution determined the number of viable counts after exposure to different treatments (Goh et al., 2021).

#### 2.6.2 Tolerance to simulated human GI tract

Using 0.8% NaCl, 0.02% KH_2_PO_4_, and 0.115% Na_2_HPO_4_ (w/v), phosphate buffered saline (PBS) was prepared. The pH of PBS was adjusted with 1N HCl to 2.5, 3.0, and 4.0, respectively by pH meter, then autoclaved at 121°C for 15 min to sterilize it. By adding pepsin (Sinopharm Chemical Reagent Co., Ltd.) to PBS buffer solution, the simulated gastric juice was prepared. A 0.22 mm filter membrane was used to filter-sterilize the pepsin solution before adding it to the sterilized PBS buffer solution (pH 2.5, 3.0, 4.0) at a final concentration of 3 g/L. The simulated intestinal juice was prepared by adding trypsin (Sinopharm Chemical Reagent Co., Ltd.) to PBS buffer solution. By using 1N NaOH to bring the pH of the broth to 8.0 and autoclaving it for 15 min at 121°C, the buffer solution broth was prepared. To achieve a final concentration of 1 g/L, the trypsin solution was filter-sterilized using a 0.22 mm filter membrane and added to the sterilized PBS buffer solution (pH 8.0). Total viable count of probiotic strains was determined by a pour plate method using MRS-agar after serial dilution for maximum recovery. MRS-agar plates were incubated anaerobically at 37°C for 8–12 h. Survival rate was calculated according to the following equation:

$$\text{Survival rate}\left(\%\right)=N_{1}/N_{0}\times 100$$

Where N_0_ is the number of viable cells after inoculation and N_1_ is the number of viable cells after treatment with artificial simulated gastric juice (8 h) or artificial simulated intestinal juice (12 h).

#### 2.6.3 Cell surface hydrophobicity analysis

The hydrophobicity of the cell surface of *Lactobacillus pentosus* (*SYBC-MI*) was evaluated using the reported method with some modifications (Wang and Li, 2022). In MRS broth, bacteria were grown at 37°C for 20 h, and then centrifuged at 1500rpm for 20 min. After washing and resuspending the pellets in PBS buffer (pH 7.4), the optical density (OD_600)_ was determined to be about 10^8^ CFU/mL. An equal volume of xylene was added and incubated at 37°C for 10 min and after that suspension was vortexed for 2 min. A two-phase system was incubated at 37°C for 1 h. The aqueous phase was determined by measuring the OD at 600 nm (A1). Using the following formula, we calculated the percentage of bacteria adhering to solvent: (A1 − A0)/A0 × 100.

#### 2.6.4 Analysis of antagonistic property

The most important property of probiotics is their ability to antagonize pathogens. The antibacterial activity was evaluated using the well diffusion method using neutralized cell-free supernatant (CFS; Kumar and Kumar, 2015). Isolated LAB were grown in MRS broth for 24 h at 37°C. Centrifugation was done to extract the cells at 14,000 g for 5 min and the supernatant was filtered through a sterile 0.22 m syringe filter. *Escherichia coli ATCC25922*, *Staphylococcus aureus ATCC29213*, and *Salmonella typhimurium ATCC14028* were three distinct pathogenic bacteria that were utilized as indicator strains and were cultivated in LB broth at 37°C for 24 h (Poornachandra Rao et al., 2015; Chuttong et al., 2016). The Luria-Bertani (LB) agar medium was produced on a Petri dish and the bacterial suspension (10^8^ CFU/mL) was evenly spaced. The Petri plates containing the Oxford cups were incubated at 37°C for 18 h to evaluate the antibacterial activity. Triplicates were performed and the diameter (mm) of the inhibition zone was then measured and expressed (Mean standard deviation).

#### 2.6.5 Auto-aggregative ability

*Lactobacillus* strain S4 was inoculated into MRS liquid medium, incubated overnight at 37°C, and re-suspended in phosphate buffer solution (pH 7.0). The turbidity was adjusted and standardized to an OD_600_ value of 0.6 ± 0.02, and the absorbance value (A_0_) was recorded accurately. A bacterial suspension in 4 milliliters (4 mL) was vortexed for 10 s and then incubated for 3 hours at 37°C. The absorbance reading of the upper portion was measured. The auto-aggregation percentage was expressed as H% = [1—A_t_/A_0_] ×100, where A0 indicates the absorbance at time 0 h and At indicates the absorbance every hour (Al-Hatamleh et al., 2020).

#### 2.6.6 Antibiotic susceptibility test

The selected *Lactobacillus* strains were tested for antibiotic susceptibility using a disc diffusion method by Clinical and Laboratory Standard Institute (CLSI) performance standards for antimicrobial susceptibility testing. Gentamycin (GEN, 10 g), Streptomycin (S, 10 g), Kanamycin (K, 30 g), Erythromycin (ERY, 15 g), Chloramphenicol (C, 30 g), Ampicillin (AMP, 10 g), and Vancomycin (VA, 30 g) were the seven different antibiotics used (Zulkhairi Amin et al., 2018; Zulkhairi Amin et al., 2020). 0.1 mL of the isolate (10^8^ CFU/mL) was applied to MRS agar plates with a sterile cotton swab. After a 24-h incubation at 37°C, the inoculum obtained from the LAB strains’ overnight culture was roughly diluted to 0.06 at OD_600_, which is equivalent to the McFarland standard 0.5, then added to each well. Following a 24-h incubation period at the ideal temperature for each LAB strain, the minimal inhibitory concentration (MIC) for growth in a microplate reader at OD_600_ was calculated in triplicate. The lowest antibiotic concentration (MIC) that completely inhibits bacterial growth was established, and an OD of 0.02 was regarded as transparent.

#### 2.6.7 Hemolysis test

The strains were streaked onto TSA media containing 5% sheep blood and cultured at 37°C for 48 h to test hemolytic activity. A clear zone (-hemolysis), a greenish zone (-hemolysis), or no zone (-hemolysis) forming around the colonies were observed.

#### 2.6.8 Ability of strain to degrade nitrite

The residual nitrite content of seven strains was measured after 48 h of incubation at 37°C in an MRS liquid medium containing 125 μg-mL^-1^ nitrite. The determination of nitrite content was performed using the national standard operating method (naphthylenediamine hydrochloride spectrophotometric method) (Sallan et al., 2023).

### 2.7 Fermentation capacity of strain

A 2% inoculum of each activated strain was added independently to an MRS liquid medium having a 10 g/L glucose concentration. The fermentation liquid’s pH value and lactic acid content were determined after 24 h of shaking at 30°C and 80 rpm. Using a Hitachi High-Performance Liquid Chromatograph with a UV absorption detector column, lactic acids were detected (Mao et al., 2020). Ion chromatography was performed using an organic acid column with a mobile phase of 5 mM sulfuric acid solution at a flow rate of 0.6 ml/min and a column temperature of 50°C. The concentrations of different lactic acids were calculated based on peak timings and peak areas. The yield was determined by subtracting the lactic acid concentration at 0 h from the lactic acid content at 24 h (Zhao et al., 2018).

### 2.8 Fermentation of millet by isolated strains

Millet grain and millet powder was prepared as a fermentation medium at a feed to liquid ratio of 1:20 (v/v) with reference to previous studies (Motahari et al., 2017). The activated strain was washed off the residue of the original medium after low temperature centrifugation and suspended with sterile saline to prepare a fermentative agent, which was inoculated into the sterilized millet fermentation medium at a 5% inoculum (v/v; Osman, 2011). During the fermentation process, samples were taken at 0 d, 2 d, 4 d, 6 d, 8 d, and 10 d to check the changes of biomass. The biomass changes in the fermentation broth with the change of fermentation time were measured by the plate colony counting method; the acidity changes during the fermentation process were detected by a pH meter. The whole fermentation experiment was repeated three times to take the average value.

### 2.9 Analysis of different amino acids in the fermentation process using HPLC

*Lactobacillus pentosus* (S1–7) strains isolated from sour honey were applied to foxtail millet fermentation, and the concentrations of most free amino acids increased with fermentation time compared to the characteristics of the initial foxtail millet fermentation broth. Analytical techniques were utilized to determine the concentrations of the amino acids tryptophan, serine, glutamate, and glutamine. To obtain the samples, shake flasks were collected and subsequently centrifuged. High-performance liquid chromatography (HPLC) with UV detection was used for this purpose. HPLC was used to determine the amino acid concentrations, using Zorbax Eclipse-AAA columns on an Agilent 1100 HPLC system following the provided instructions. This specific column had a resolution ratio of approximately 10pmols. Manufacturer’s protocol was followed for the derivatization process. The amino acids tryptophan, serine, glutamate, and glutamine were separated using a mobile phase consisting of phase A (40 mM Na_2_HPO4, pH 7.8) and phase B (Acetonitrile (ACN). Glucose, acetate, and α-ketoglutarate were quantitatively analyzed using an Aminex HPX column at a column temperature of 30°C, a flow rate of 2 mL/min, and a measure wavelength of 350 nm. All experiments were conducted in triplicate, and one sample was analyzed within a 30-min timeframe.

### 2.10 Analysis of tryptophan accumulation

*Lactobacillus pentosus S4*, the most acid-producing bacterium, was used as an indicator strain for exploring fermentation conditions. Further, *Lactobacillus pentosus S4* was fermented at 30°C for millet powder and millet grains in MRS medium, respectively, and the changes of L-tryptophan content in the fermentation broth were observed by UV spectrophotometer.

### 2.11 Statistical analysis

All data are the mean of three parallel experimental measurements. Significance analysis between data was performed using the one-way ANOVA method with SPSS^®^ 21.0 (IBM, USA) at a significance level of *p* ≤ 0.05.

## 3 Results

### 3.1 Bacterial diversity and sequence analysis of stingless bee honey

A total of 318,346 high-quality sequences were generated, with an average of 79,587 sequences per sample, by the high-throughput sequencing of sour honey samples collected from four different habitats in Yunnan, China. The bacterial OTU number detected in sample Y1 was 276, the largest among the four samples. The Coverage index of the four samples was greater than 0.9997, indicating that the coverage of the sample library was high and the possibility of undetected sequences was small. Thus, the constructed library could accurately reflect the microbial diversity of the samples to be tested. ACE, Chao1, Shannon, and Simpson indices were used to evaluate the alpha diversity among the bacterial communities of the four species found in stingless bee honey. As shown in Table 2, according to the average Shannon indices and average Simpson indices of bacteria in the four kinds of sour honey samples, the highest abundance of bacteria was found in sample Y3, followed by Y1. The rank abundance curve of sample Y1 was the flattest, which directly indicates that sample Y1 had the highest uniformity of species composition, and the steepness of sample Y4’s curve was the greatest, indicating that sample Y4 had the least diverse species composition (Supplementary Figure S1).

**TABLE 2:** Estimations of the abundance and diversity levels of bacterial communities in four different sour honey samples (Y1–4).

Sample	Reads	OTUs	ACE	Chao1	Simpson	Shannon	Coverage
Y1	79587	276	278.06	277.24	0.83	3.57	0.9999
Y2	79672	271	277.88	280.05	0.53	2.27	0.9998
Y3	79551	254	260.17	260.18	0.89	3.76	0.9999
Y4	79536	257	285.81	304.22	0.54	2.13	0.9997

**FIGURE 1:**
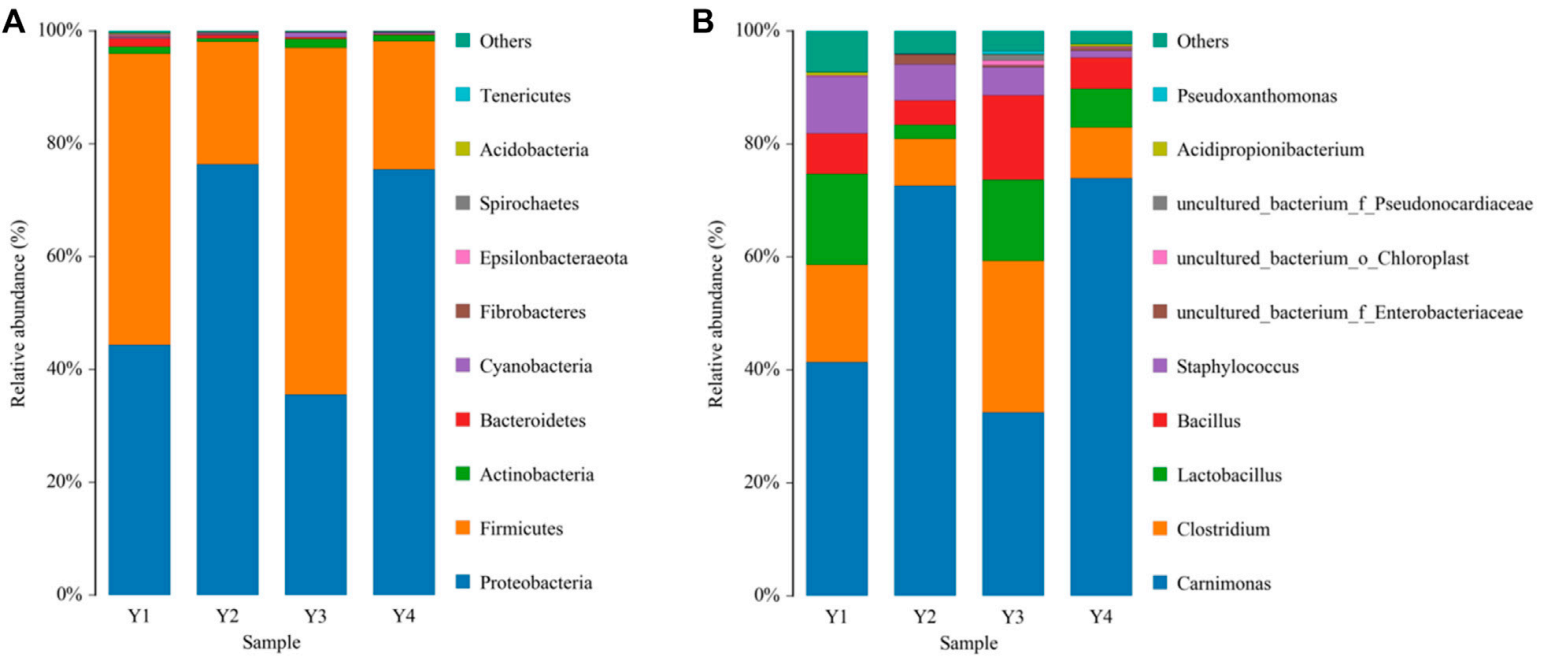
Bacterial compositions phylum **(A)** and genus **(B)** levels in four different sour honey samples (Y1–4). The color-coded bar graphs show the average bacterial phylum and genus distributions for the different groups sampled from the four sour honey samples.

Most OTUs were identified as Firmicutes and Proteobacteria at the phylum level, the main bacterial groups’ common to all four sour honey samples, but with slightly different proportions. The relative abundance of *Proteobacteria* was 44.58%, 76.66%, 35.63%, and 75.72%, respectively. *Firmicutes* were more abundant in Y1 (51.89%) and Y3 (61.59%) but less abundant in Y2 (21.77%) and Y4 (22.75%), and the relative abundances of other phyla accounted for approximately 2% Figure 1A. In addition, OTUs ranked in the top five dominant genera at a bacterial level among the four sour honey samples, and *Carnimonas* had the highest relative abundance, accounting for 74.75% in Y2 and 75.29% in Y4, making it the dominant genus in the two sour honey samples. *Carnimonas* accounted for 43.41% in Y1 and 33.36% in Y3. This was followed by *Clostridium*, which reached 27.60% in Y3. The relative abundance levels of *Lactobacillus* varied significantly among the four types of sour honey at 16.90%, 2.58%, 14.72%, and 6.97%, respectively. *Lactobacillus* was abundant in Y1 and Y3, and *Bacillus* accounted for 7.45%, 4.41%, 15.33%, and 5.58%, respectively. *Staphylococcus* accounted for 10.53%, 6.58%, 5.13%, and 1.31%, respectively. In addition, *Pseudoxanthomonas* and *Acidipropionibacterium* were also present Figure 1B.

**FIGURE 2:**
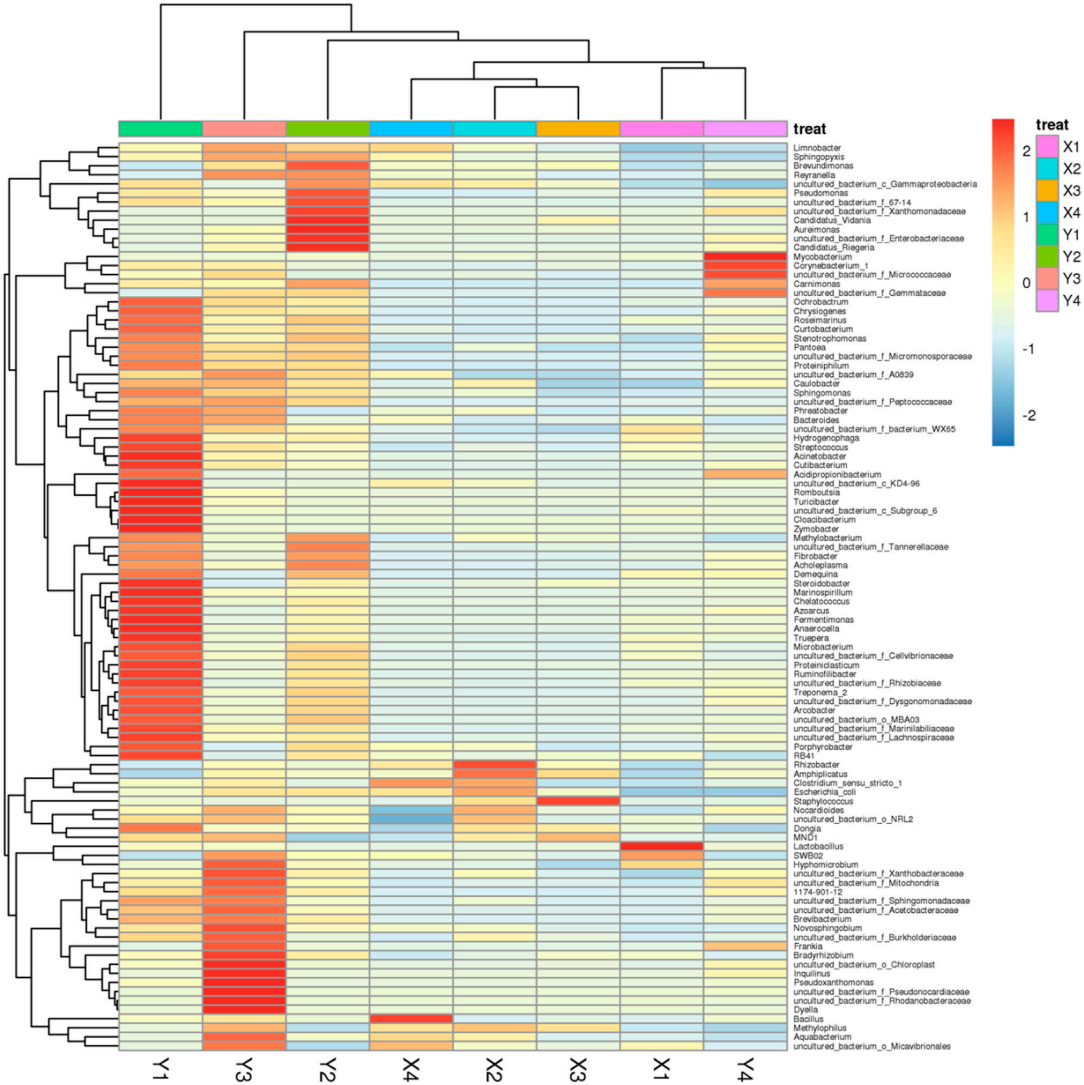
Species abundance Heat map showing the community composition of sour honey samples; its similarities and variances.

However, after the domestication of sour honey by MRS medium, the main species among them changed to the phylum Firmicutes, and their relative abundance all exceeded 98.5%, which was significantly increased compared to the pre-domestication period. There was a significant change in species abundance for each acid honey sample before and after domestication. After domestication of acid honey by MRS medium, all S*arcocystis spp. (Carnimonas)* decreased substantially to less than 0.5%. Each sample species had a different set of bacterial abundance (Y1, 52 genera; Y2, 17 genera; Y3, 32 genera; Y4, 7 genera. Sample Y1 had the greatest abundance of bacterial genera. The community composition of several samples of sour honey and its similarities and variances were demonstrated by a species abundance heat map (Heat map), as shown in Figure 2. The bacterial structures in the acid honey samples from four different production areas were largely similar, but there were some differences in bacterial diversity and bacterial species abundance levels, which may be related to the different floras in different habitats from which the acid bees were collected. In addition, acidity is also an important factor that may affect bacterial diversity in sour honey (Rosli et al., 2020).

### 3.2 Identification of novel acid-resistant strain S4

**FIGURE 3:**
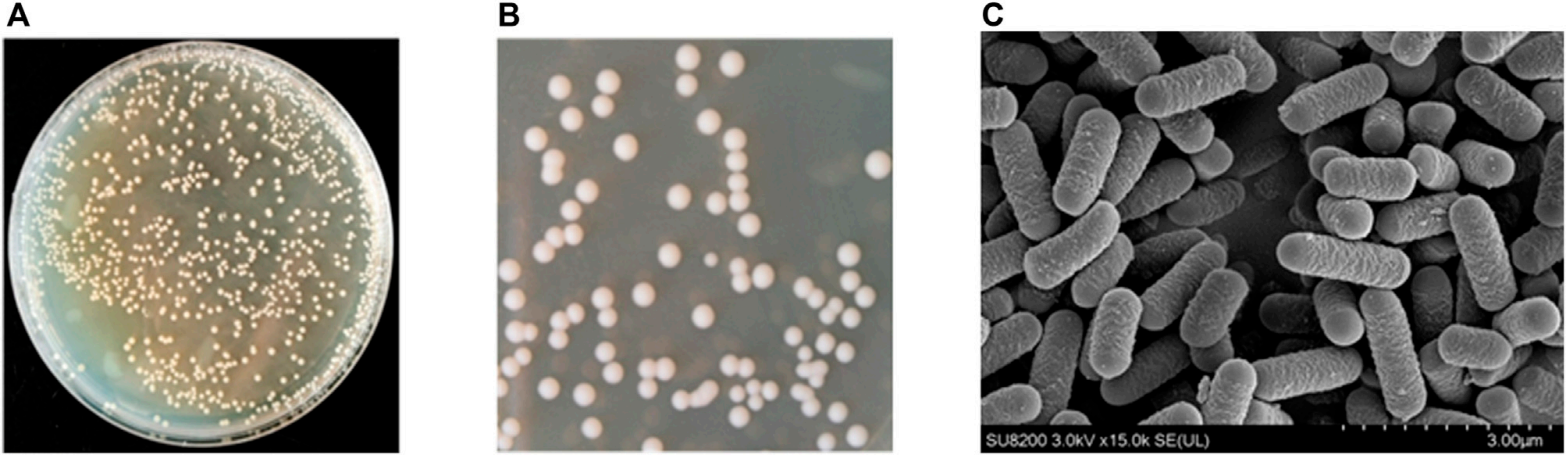
**(A)** Lactic acid production in 48 h of anaerobic culturing, colonies with diameters of 0.2–2.1 mm growing on the MRS agar containing CaCO_3_ **(B)** Observation of colony morphology of the isolates, which was seen to be creamy white, sub-circular, about 2–3 mm in diameter, with a moist, opaque, elevated surface, with neat edges and a uniform texture **(C)** Field emission scanning electron microscope images of *Lactobacillus* sp. S4 with magnification of ×15,000.

According to the above results, the genus *Lactobacillus* exists in sour honey; consequently, we used multiple rounds of enrichment culturing to isolate and identify *Lactobacillus* spp. from sample Y1, which had the highest relative abundance of the genus *Lactobacillus*. After 48 h of anaerobic culturing, many colonies, with diameters of 0.2–2.1 mm, grew on the MRS agar separation medium containing CaCO_3_ (Figure 3A). Single colonies with transparent circles were selected and streaked on plates to make pure culture. The single colonies with microscopic observation matching the characteristics of *Lactobacillus* were picked and re-screened on MRS plates, and incubated upside down at 37°C for 48 h. Figure 3B depicts the observed colony morphology of the isolates, which was seen to be creamy white, sub-circular, around 2–3 mm in diameter, with a wet, opaque, raised surface, with tidy edges and a uniform texture. The obtained pure cultures were subjected to gram staining, and seven positive strains were obtained. They were coded as S1–7. The colonies of these seven isolated strains were similar in morphology, with diameters of 0.5–2.0 mm, white, neat edges, ridges, and smooth and wet surfaces. From the preliminary observations, these seven isolates were considered lactic acid bacteria. Biochemical tests and molecular identification (16SrRNA sequencing) was performed to identify and characterize the novel acid resistance strain S4 (Section 3.3). Furthermore, the FESEM image for *Lactobacillus* spp. S4 (Figure 3C) showed it to be rod-shaped, with lengths ranging from 1.11 μm to 1.67μm, without flagella, and the same for the other *Lactobacillus* strains obtained by isolation.

### 3.3 Physiological and biochemical comparison of S4 with other related strains

As shown in Table 3, that all seven isolates were negative for peroxidase activity, all were unable to produce H_2_S, unable to produce indole, unable to liquefy gelatin nor were they motile. When the NaCl concentration in the medium was 6%, *Lactobacillus* spp. S1 and *Lactobacillus* sp. S4–6 survived normally. *Lactobacillus* spp. S1 and *Lactobacillus* spp. S3–5 had weak reactions when the NaCl concentration was 8%, and the other strains did not grow. In addition, except *Lactobacillus* spp. S4, the other strains did not grow at a 10% NaCl concentration. A series of tests were conducted to determine the characteristics and sugar fermentation modes of the seven *Lactobacillus* strains. A carbohydrate fermentation analysis is usually performed on newly discovered LAB to determine their fermentation capacities and to identify which carbohydrate substrates are not fermentable by the LAB strain. Here, the seven LAB strains with only slight differences were assimilated by 14 substrates, excluding L-rhamnose, L-xylose, L-sorbose, and inositol.

**TABLE 3:** The characteristics and carbohydrate fermentation patterns of seven *Lactobacillus* strains.

Characteristic	Isolated strains						
	S1	S2	S3	S4	S5	S6	S7
Growth in MRS at							
15°C	−	w	−	−	w	w	−
45°C	−	−	−	−	−	−	−
The Optimum growth temperature (°C)	30	30	30	30	35	30	30
pH 4.0	++	++	++	++	++	++	++
pH 3.5	+	+	+	+	+	+	+
pH 3.0	−	+	w	+	+	−	+
pH 2.5	−	−	w	+	−	−	−
pH 2.0	−	−	−	−	−	−	−
Growth in MRS with NaCl at							
6%	+	w	w	+	+	+	W
8%	w	−	w	w	w	−	−
10%	−	−	−	−	w	−	−
Others							
Motility	−	−	−	−	−	−	−
Gram stain	+	+	+	+	+	+	+
Catalase assay	−	−	−	−	−	−	−
H_2_S production test	−	−	−	−	−	−	−
Gelatin liquefaction test	−	−	−	−	−	−	−
Indole production test	−	−	−	−	−	−	−
Starch hydrolysis test	+	+	+	+	+	+	+
Acid production from							
L-arabinose	+	+	+	+	+	+	+
D-cellulose-disaccharide	+	+	+	+	+	+	+
D-galactose	+	+	+	+	+	+	+
D-lactose	++	++	++	++	++	++	++
D-maltose	+	++	++	++	++	+	++
D-mannose	+	++	+	++	++	+	++
D-mannitol	+	+	+	+	+	+	+
D-triose	−	+	−	−	+	−	−
L-rhamnose	−	−	−	−	−	−	−
D-ribose	+	+	+	+	+	+	+
D-sucrose	+	+	+	+	+	+	+
D-xylose	−	−	−	+	−	−	−
L-xylose	−	−	−	−	−	−	−
D-fructose	+	+	+	+	+	+	+
D-sorbitol	+	+	+	+	+	+	+
L-sorbose	−	−	−	−	−	−	−
inositol	−	−	−	−	−	−	−
glycerol	+	+	+	+	+	+	+

++, strong positive; +, positive; w, weak positive; −, negative.

### 3.4 Genome characteristics of strain S4 (*Lactobacillus pentosus SYBC M1*)

#### 3.4.1 Genome prediction

Whole genome sequencing analysis was performed on *Lactobacillus pentosus* strain S4, a strain found to have potential acid resistance ability, degrade nitrite and accumulate tryptophan in millet fermentation applications; hence, this strain was named *Lactobacillus pentosus SYBC-M1.* The gene prediction results of this strain are shown in Table 4. The complete genome of *L. pentosus SYBC- M1* consists of a circular chromosome (3,288615 bp; Supplementary Figure S2) with plasmids of 11,466 bp lengths. The GC content is 44.03%. The number of predicted genes is 3,129, with 16 rRNAs and 74 tRNAs present. The tRNAscan-SE software can predict the tRNA region and secondary structure of tRNAs. The software identifies 99%–100% of tRNA genes with a sensitivity of less than one false positive per 15 kilobases, making it the most widely used and beneficial tool for predicting tRNAs. It is the most widely used and beneficial tool for tRNA prediction. The CRISPR finder was used to predict the genome’s DRs (direct repeated sequence) and spacers (spacer regions). Zero CRISPR structures were predicted in the sample MTT.

**TABLE 4:** Genome prediction of *Lactobacillus pentosus* SYBC-M1.

Type	Quantity
Genome length	3,287,615
Longest bp	152,906
Shortest bp	200
Protein coding genes	3,129
GC content %	44.77
tRNA	74
N50 (bp)	17,228
N20 (bp)	38,987
N90 (bp)	2,823
Q20%	97.86
Q30%	93.73

#### 3.4.2 Genome functional annotation

The genomic functional annotation of S4 (OM618128; *Lactobacillus pentosus SYBC-M1*) was annotated through the KEGG (Kyoto Encyclopedia of Genes and Genomes) GO, KEGG, NR, CAZy, CARD. Swiss-Prot and Pfam databases (blastp, evalue 1 105, identity 40%, and coverage 40%) were used to understand its genes’ biological roles further. The final annotation values are shown in Supplementary Table S1. The statistics of the KEGG Pathway with chr (cell cycle genes homology region) sequences are shown in Supplementary Figure S3. Among the biological pathways corresponding to the predicted genes of strain S4. Protein families: genetic information processing (454), signaling and cellular processes (361), metabolism (171), amino acid metabolism (159), membrane transport (150), and carbohydrate metabolism (291) had the highest number of annotated genes. In addition, 82 genes were involved in the Energy metabolism pathway, 81 genes in the Metabolism of cofactors and vitamins pathway, 66 genes were annotated to be involved in the Nucleotide metabolism pathway, and 56 genes in the Nucleotide metabolism pathway. Most genes associated with amino acid, carbohydrate, and membrane transport have been found. The strain’s capacity to survive in various environments can be observed by genes involved in acquiring and utilizing nutrients. Similarly, GO (Gene Ontology) was developed to address uncertainties in the definition of the same gene across databases and difficulties in functional characterizing the same gene between species. The results of the GOSlim analysis are shown in Supplementary Figure S4.

The results of prophage prediction showed that the chr (cell cycle genes homology region) sequence predicted six prophages in the genome (Supplementary Table S2). Prophage (prophage) refers to integrating nucleic acids of certain mild phages into the host bacterial chromosome after infesting a bacterium. Secretory protein sequences existed in 76 protein-coding genes in the chr sequence, accounting for 2.43% of the total number of protein-coding genes; 0 protein-coding genes existed in the plasmid1 sequence, accounting for 2.43% of the total number of protein-coding genes. The secreted protein sequences were present in protein-coding genes in the plasmid1 sequence, accounting for 0% of the total protein-coding genes (Supplementary Table S3).

The hmmscan software was used to predict the presence of CAZy enzyme-like genes in the genome sequence (Supplementary Figure S5). ORF sequences > 80 amino acids with E-value threshold set at 1e-5 and amino acid sequences > 30% of the amino acid sequences in the database were selected; sequences < 80 amino acids with E-value threshold set at 1e-3 and amino acid sequences > 30% of the amino acid sequences in the database were also selected. The database contains families of enzymes related to glycosidic bond degradation, modification and production. It contains five main categories: Glycoside Hydrolases (GHs), Glycosyl Transferases (GTs), Polysaccharide Lyases (PLs), Carbohydrate Esterases (CEs), and Auxiliary Activities (AAs; Supplementary Figure S5).

Similarly, The BLAST software was used to predict the genes associated with antibiotic resistance in the genome, the BLAST alignment parameter E-value was set at 1e-6, and the amino acid sequence identity was above 45%. The E-value was set at 1e-6, the amino acid sequence identity was above 45%, and the ratio of the length of the sequence alignment to the length of the sequence was not less than 70%. The antibiotic resistance analysis results show that 25 genes have been identified, with 19 genes for antibiotic resistance, 11 for antibiotic target, and 1 for antibiotic biosynthesis (Supplementary Table S4). The LAB strains isolated from sour honey were susceptible to gentamicin, erythromycin, chloramphenicol, ampicillin, and vancomycin just like the majority of probiotic bacteria. Antibiotic LAB strains’ intrinsic resistance is not regarded as a threat to both animal and human health.

#### 3.4.3 Phylogenetic analysis

**FIGURE 4:**
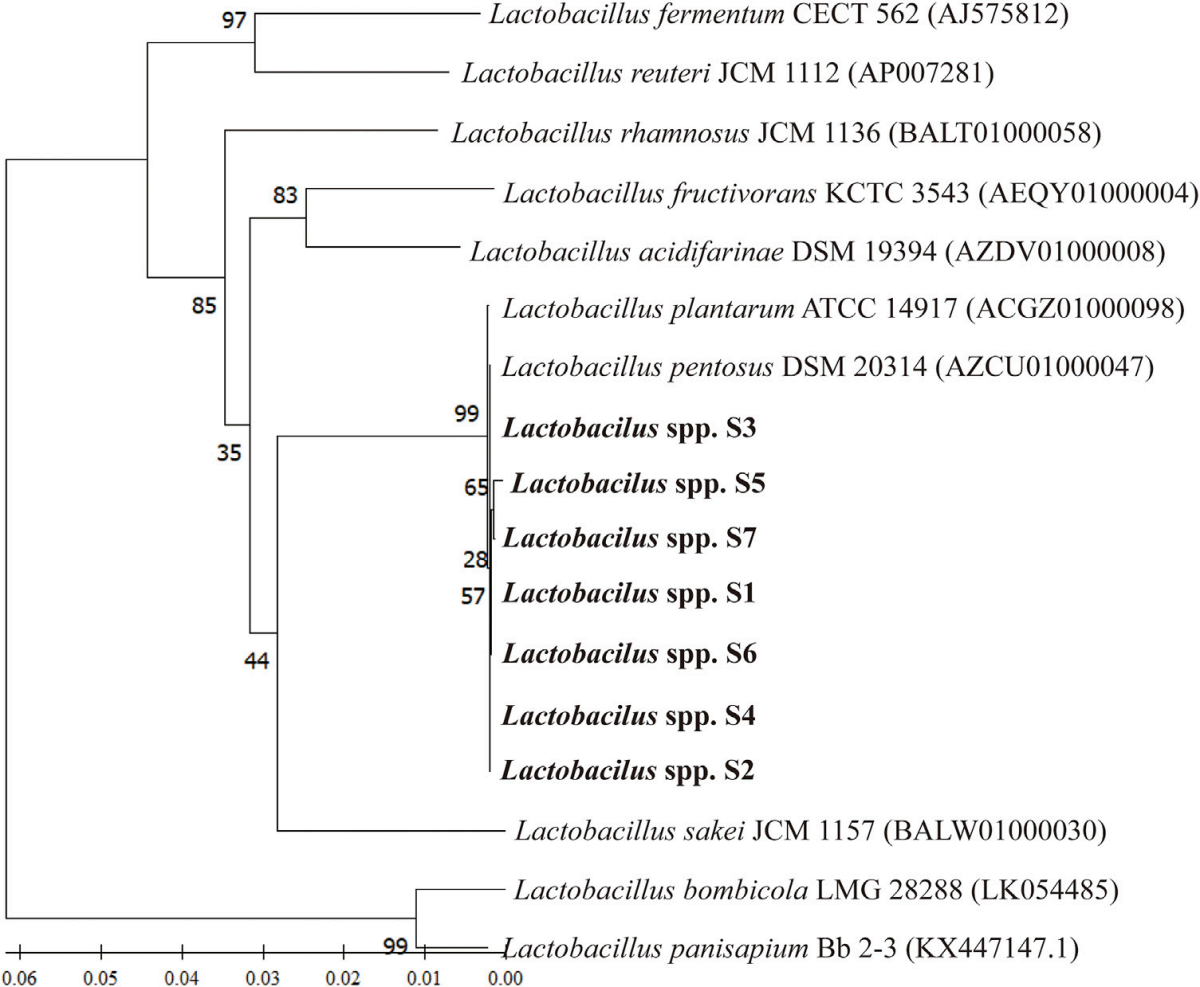
The phylogenetic relationship between selected *Lactobacillus* strains and *L. pentosus SYB-MI* with the neighbor-joining approach and the 16S rRNA gene.

The phylogenetic tree constructed using 16S rRNA gene sequences and neighbor-joining methods revealed the relationships between the seven *Lactobacillus* spp. strains and their phylogenetically closed relatives. The robustness of the topology of the phylogenetic trees was evaluated by a bootstrap analysis based on 1,000 replications. All seven *Lactobacillus* strains represented a separate lineage within the *Lactobacillus* genus, including *Lactobacillus pentosus* (Figure 4). The results of a sequence alignment using BLASTn showed that all seven *Lactobacillus* strains were most similar to *L. pentosus*, with similarity scores of 99.47%, 99.79%, 99.89%, 99.26%, 99.47%, 99.47%, and 99.37%, respectively.

### 3.5 *In vitro* tests for the probiotic potential of acid-resistant *Lactobacillus* spp. S4

#### 3.5.1 Resistance to simulated gastric juice and bile salts

**FIGURE 5:**
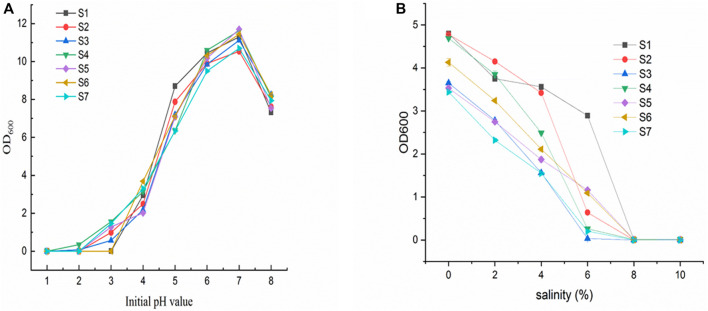
**(A)** Growth of isolates (S1–7) at different initial pH; **(B)** Growth of isolates (S1–7) at different salt concentrations.

To simulate conditions in the stomach, we examined the ability of the LAB isolates to survive in artificial gastric juice at pH 2.5 and temperatures of 15°C and 45°C for 48 h. The S2, -5, and -6 strains grew weakly at 15°C, but none of the seven isolates grew at 45°C. All the strains grew well at pH 4.0, and they all grew normally at pH 3.5. Strains S2, -4, -5, and -7 grew at pH 3.0, whereas only strain S4 grew at pH 2.5. None of the strains grew at pH 2.0. The best growth temperature for all other strains, except strain S5, was 30°C for 48 h. Strain S5 grew at 35°C. Therefore, only one strain, S4, demonstrated tolerance to pH 2.5 for 4 h during the screening of acidity-resistant bacteria. We assessed the ability of the LAB isolates to survive in an artificial gastric juice at pH 2.5, simulating conditions similar to those in the stomach, using an acid and bile tolerance experiment Figure 5A.

Figure 5B shows the effect of different salt concentrations on the growth of the isolates (S1-7). All strains have an appropriate level of salt tolerance. In the presence of 0.3% bile, four isolates (S1, S2, S4, and S6) demonstrated the strongest bile resistance (over 50% compared to growth control). On the other hand, S3 and S7 had the highest survival rates in the presence of 0.5% and 1% bile (28% and 19%, respectively).

#### 3.5.2 Hydrophobicity and auto aggregation

An important indicator of probiotic adhesion to the intestine is hydrophobicity and auto-aggregation. As seen in Table 5 after 24 h of incubation the hydrophobicity capacity of was S4 was 28.0428.04% ± 1.30%. Additionally, the auto-aggregation capacity was 25.06% ± 1.41%.

**TABLE 5:** Tolerance to artificial simulated gastrointestinal conditions and cell surface hydrophobivity of *L. pentosu*s SYBC-MI.

Strain	Artificial simulated gastric juice		Cell surface hydrophobicity
	8 h survival rate %	12 h survival rate %	
S4	78 ± 3.66^ab^	70 ± 4.49^ab^	28.05 ± 1.32

^a,b^: Different superscript letters in the same column indicate statistical differences at the level of *p* < 0.05. Values are represented as mean ± SEM.

#### 3.5.3 Nitrite degradation ability

The isolated *Lactobacillus* strain S4 was cultured in MRS liquid medium containing 125 μg-mL^-1^ nitrite. The residual nitrite content was measured after 48 h incubation. The results are shown in Table 6, this isolate had a high ability to degrade nitrite, exceeding 99.5%, and the final pH of the culture solution was around 4-5.

**TABLE 6:** Ability of the isolated strains to degrade nitrite.

Strain number	Final nitrite content (μg·mL^-1^)	Degradation rate (%)	Final pH value
S1	0.27	95.78	4.01
S2	0.38	96.70	4.03
S3	0.36	96.71	4.03
S4	0.46	99.71	4.04
S5	0.36	98.71	4.01
S6	0.30	97.68	3.93
S7	0.35	96.72	4.02

#### 3.5.4 Antibacterial and sensitivity testing

Seven lactic acid bacteria were tested for their antibacterial efficacy. Strain “S4” was selected for the next round of studies because it has the strongest antibacterial activity among the seven bacteria listed in Table 7; based on the bacteriostasis of each strain on *S. Typhimurium, E. coli,* and *S. aureus;* additionally, the outcomes showed that the *L. pentosus* strain S4 had excellent probiotic properties. Sensitivity testing of several commonly used antibiotics showed that strain S4 was sensitive to gentamicin, erythromycin, chloramphenicol, ampicillin, and vancomycin; and resistant to streptomycin and kanamycin. This further validates the safety of its use as a potential probiotics (Table 7).

**TABLE 7:** *In vitro* tests for probiotic potential of *Lactobacillus* sp. S4.

Strain	Indicator organism (mm)			Susceptibility to antibiotics MIC (µg/L							Auto-aggregation
	*E. coli*	*Salmonella*	*S. aureus*	GEN	S	K	ERY	C	AMP	VA	
S4	12.69 ± 0.41	−	10.7 ± 0.21	I	R	R	S	S	S	R	25.06 ± 1.41
				2	0.25	128	8	4	0.5	0.06	

Results were expressed in mm (mean ± SD) of three measurements.

MIC values expressed as μg mL^−1^ for the evaluation of antibiotic resistance of the *Lactobacillus* strains.

R, resistant; S, susceptible; I, intermediate.

#### 3.5.5 Hemolytic activity of *L. pentosus* SYBC-MI

It was determined that isolates with a clear halo were β-hemolytic, and isolates with a green halo were designated as α-hemolytic, isolates presenting no halo were classified as γ-hemolytic. In our study *L. pentosus* demonstrates γ–hemolysis (Supplementary Figure S6).

### 3.6 Fermentation test profile

**FIGURE 6:**
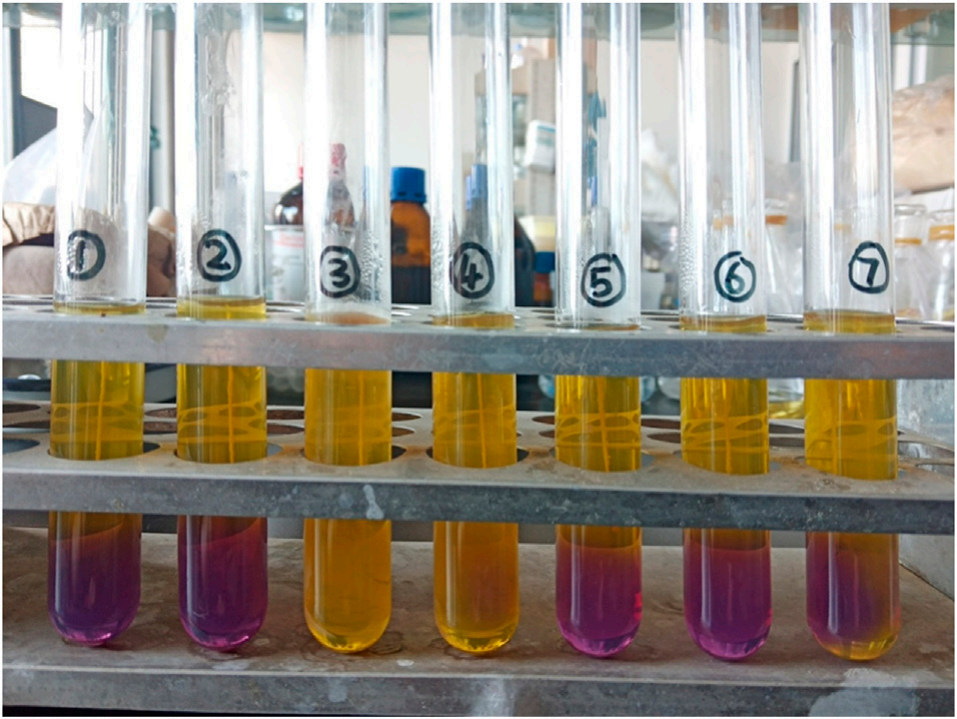
Acid and gas production tests using glucose fermentation were performed with the seven *Lactobacillus* strains to distinguish the type of LAB fermentation, homo-fermentative or hetero-fermentative. A color changed from purple to yellow indicated that the strain produced acid.

**FIGURE 7:**
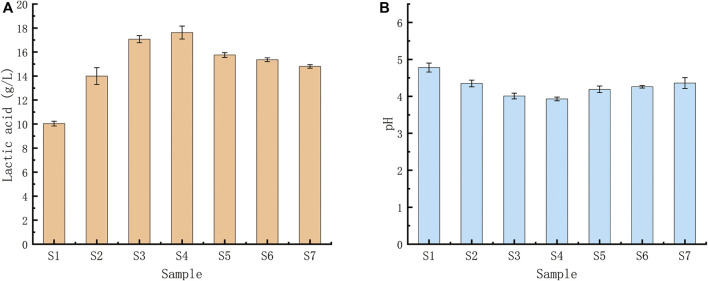
**(A)** Acid production capacities of seven *Lactobacillus* strains using glucose fermentation. **(A)** Lactic acid yields of the seven *Lactobacillus* strains after a 24-h fermentation; **(B)** pH levels of the fermentation broths after 24 h. *Lactobacillus* sp. S4 produced the highest amount of lactic acid and the lowest pH, followed by *Lactobacillus* sp.

None of the seven LAB strains produced CO_2_, and the fermentation type was homo-fermentative (Figure 6). High-performance liquid chromatography was used to analyze the yield of lactic acid, the seven LAB strains’ main fermentation product. The yields of lactic acid fermentation of seven strains (S1-S7) after 24 h were 10.04 (±0.04), 14.00 (±), 17.07 (±0.07), 17.62 (±0.14), 15.75 (±0.09), 15.36 (±0.12) and 14.80 (±0.07) g/L, respectively. *Lactobacillus* sp. S4 produced the highest lactic acid and the lowest pH, followed by *Lactobacillus* sp. S3 (Figure 7).

### 3.7 Fermentation of millet by isolated strains

In the process of fermentation of millet, it is important to use lactic acid bacteria that can produce acid quickly in a short period. This is because lactic acid bacteria with high acid-producing capacity can rapidly lower the pH of the fermentation broth, thereby inhibiting the growth of other microorganisms and resulting in improved fermentation performance**.** Therefore, *Lactobacillus pentosus* S4, the most acid-producing bacterium, was used as an indicator strain for exploring fermentation conditions (pH, temperature).

The composition of 17 free amino acids, except tryptophan, in the millet fermentation broth at the beginning and end of fermentation (day 0 and day 10) for each *Lactobacillus pentosus* isolate fermenting millet flour was examined. As fermentation proceeded, the characteristics of free amino acids in the fermentation broth of different strains differed. The concentration of most free amino acids increased with increasing fermentation time compared to the characteristics of the initial millet fermentation broth, but the variations exhibited varied (Supplementary Table S5).

#### 3.7.1 Concentration of free amino acids in millet fermentation

The increase of threonine among essential amino acids was the most obvious, and the concentrations after 10 days of fermentation by *Lactobacillus pentosus* S1-6 were 2.09, 2.01, 3.60, 2.80, 4.92 and 3.70 times the threonine content in the initial millet fermentation broth, respectively, and the threonine content after 10 days of fermentation by *Lactobacillus pentosus* S7 was slightly reduced. In the millet fermentation broth of *Lactobacillus pentosus* S1 and S3, all essential amino acid fractions decreased except threonine, which increased with the fermentation time. The millet fermentation broth of *Lactobacillus pentosus* S2 showed the largest increase in essential amino acid fractions, and the content of eight essential amino acids at the end of fermentation was 293.41 mg/L, accounting for 30% of the total free amino acids. Among them, the concentrations of valine, methionine, isoleucine, leucine and lysine were 1.95, 2.01, 3.47, 3.14 and 1.38 respectively. The concentrations of valine and methionine were 1.67 and 3.15 times higher than those in the initial millet fermentation broth of *Lactobacillus pentosus* S4, respectively. The concentrations of valine, methionine and isoleucine were 1.71, 2.14 and 1.37 times higher, respectively, than *Lactobacillus pentosus* S6, except for threonine. Only isoleucine increased in essential amino acids, and the concentrations were 1.26 times higher than the initial millet fermentation broth, while in the millet fermentation broth of *Lactobacillus pentosus* S7, only valine and lysine increased in essential amino acids. The concentrations were 2.63 and 1.72 times higher than the concentration in the initial millet fermentation broth, respectively (Supplementary Table S5).

Among the non-essential amino acids, aspartic acid, glutamic acid, and glycine contents were significantly increased after 10 days of fermentation by *Lactobacillus pentosus* S1-6, and the concentrations of aspartic acid were 1.34, 1.11, 1.35, 1.02, 1.19, and 1.38 (mg·L^-1^) times higher than those in the initial millet fermentation broth, respectively. The concentrations of glutamic acid were 1.37, 2.26, 1.19, and 1.38, 1.33, 1.24 (mg·L^-1^) times higher and the concentration of glycine was 2.90, 1.85, 3.66, 2.39, 2.49, and 3.59 (mg·L^-1^) times of the initial millet fermentation broth, respectively; only *Lactobacillus pentosus* S7 had lower aspartic acid, glutamic acid, and glycine contents after 10 days of fermentation. In addition to the above three non-essential amino acids, only the content of serine and histidine increased in *Lactobacillus pentosus* S1 after 10 days of fermentation, and the concentrations were 3.07 and 1.45 (mg·L^-1^) times higher than those in the initial millet fermentation broth, respectively (Supplementary Table S6). The non-essential amino acid fraction increased more in the fermentation broth of *Lactobacillus pentosus* S2, and the concentrations of alanine, tyrosine, cystine, and proline were 4.04, 2.19, 1.41, and 1.01 times higher than those in the initial millet fermentation broth, respectively. The fermentation broth of *Lactobacillus pentosus* S3 and S4 showed an increase in both serine and histidine, with the difference that the tyrosine of *Lactobacillus pentosus* S3 improved, but the tyrosine of *Lactobacillus pentosus* S4 decreased. The fermentation broth of *Lactobacillus pentosus* S5 showed 1.57 and 1.10 times higher concentrations of serine and cystine, respectively, than the initial millet fermentation broth; the alanine of *Lactobacillus pentosus* S6 increased slightly. Slightly increased: *Lactobacillus pentosus* S7 fermented for 10 days, only the serine, histidine, and alanine content increased.

The increase of threonine among the essential amino acids was the most pronounced, with the concentration after 10 days of fermentation by *Lactobacillus pentosus* S1–6 being on average 3.19 times higher than the value in the initial foxtail millet fermentation broth. The essential amino acid fraction increased the most in the millet fermentation broth of *Lactobacillus pentosus* S2, with the total content of eight essential amino acids at the end of fermentation being 293.41 mg L^-1,^ accounting for 30% of the total free amino acids. In addition, the changes in taste-presenting amino acids varied. After comprehensive consideration, it was found that 6 days of fermentation was the best time for foxtail millet fermentation. When the fermentation broth of almost all isolates had the lowest content of bitter amino acids (histidine, arginine, tyrosine, valine, methionine, isoleucine, leucine and phenylalanine) and the highest content of fresh and sweet amino acids (serine, glycine, threonine, alanine, lysine and proline**)**.

#### 3.7.2 Tryptophan uptake by isolated strains during millet fermentation

**FIGURE 8:**
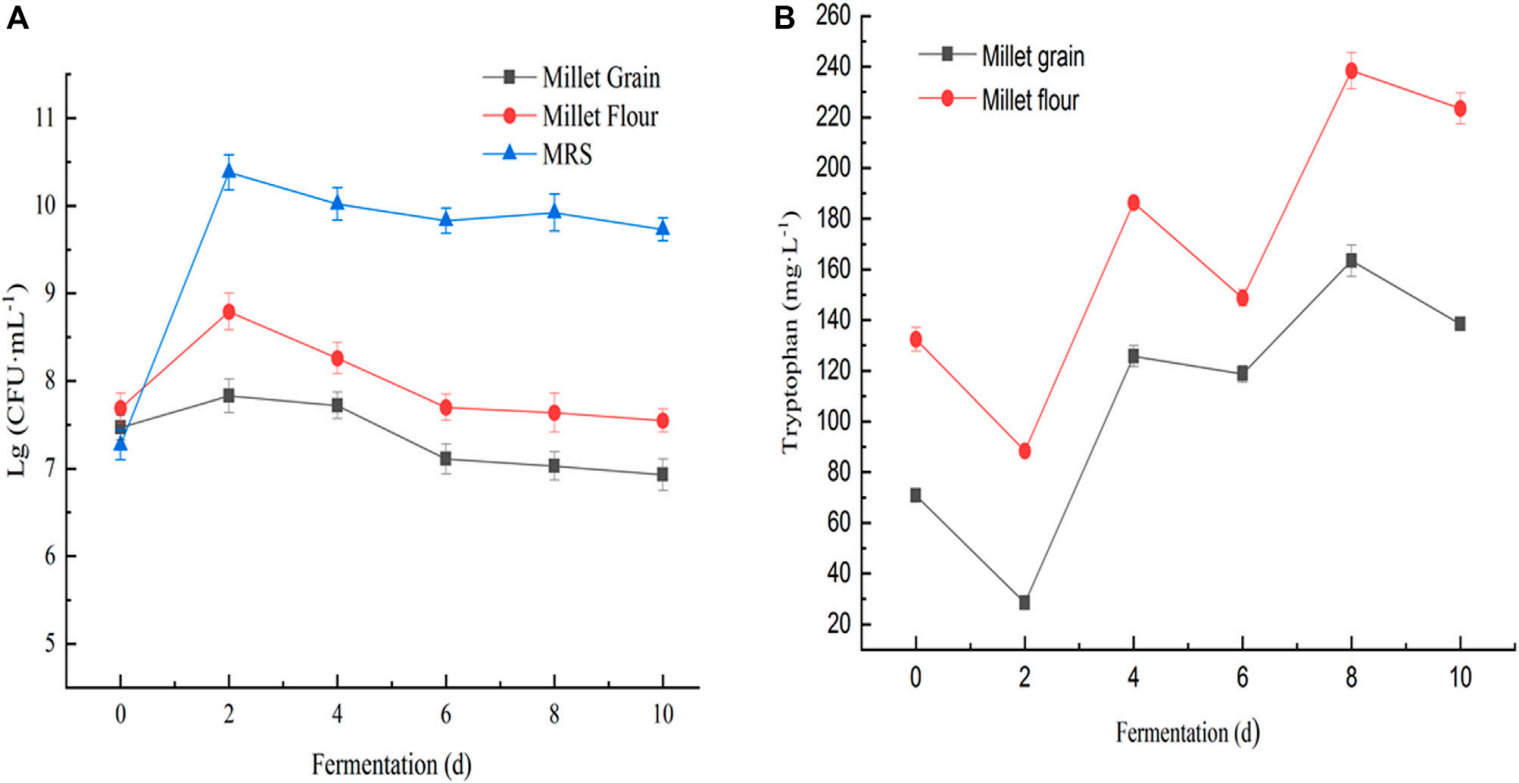
**(A)** Changes in biomass of *Lactobacillus* sp. *S4* cultured on MRS medium, millet grain medium, and millet flour fermentation medium; **(B)** Changes in tryptophan content of *Lactobacillus* sp. S4 when fermenting millet flour and millet grains at 30°C, respectively.

**FIGURE 9:**
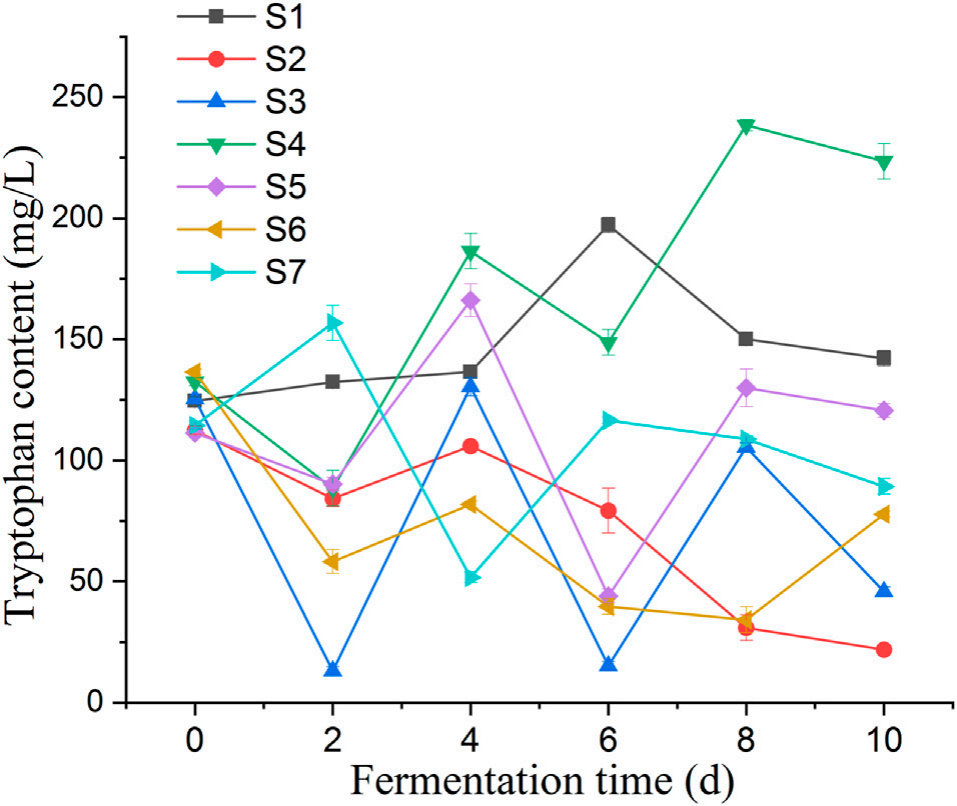
Represent the changes of tryptophan content of fermentation broth for 10 days of millet flour fermentation by *Lactobacillus pentosus S1-7*, respectively; *Lactobacillus pentosus S4* was fermented at 30°C for millet powder and millet grains, respectively, and the changes of L-tryptophan content in the fermentation broth were observed. The highest tryptophan content was found at the day 8, and the results proved that the highest tryptophan content was achieved at 30°C of fermentation temperature.

The accumulation of tryptophan in the fermentation broth of millet flour was significantly higher than that in the fermentation broth of millet grains during 10 days of millet fermentation. Changes in biomass of *Lactobacillus spp.* cultured on MRS medium, millet grain medium, and millet flour fermentation medium (Figure 8A). The initial tryptophan content in the fermentation broth during the fermentation of millet flour was higher than that of millet grain fermentation, which indicates that the amino acids and other nutrients in millet entered the fermentation medium better after grinding and homogenization. During the fermentation of foxtail millet by seven *Lactobacillus pentosus* (S1–7) strains isolated from sour honey, a potential tryptophan accumulating isolate, *Lactobacillus pentosus* S4, was obtained, which could reach a maximum tryptophan content of 238.43 mg L^-1^ in the fermentation broth, 1.80 times the initial tryptophan content in the fermentation broth (Figure 9).

#### 3.7.3 Changes of pH and L. tryptophan in millet fermented by acid honey lactobacilli

**FIGURE 10:**
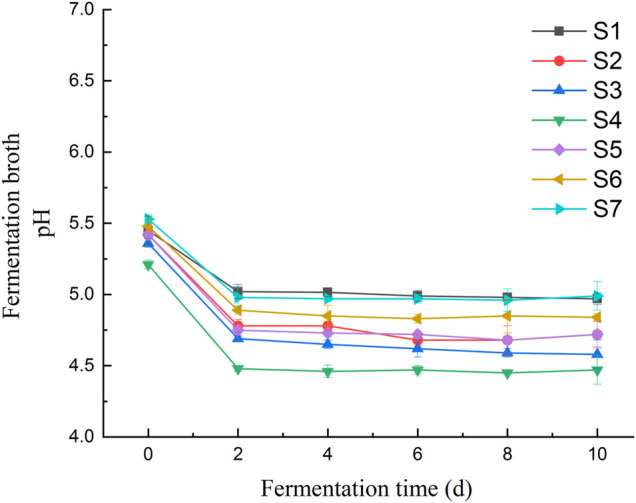
pH changes of fermentation broth for 10 days of fermentation.

Changes of pH and L-tryptophan content was observed in the fermentation broth of seven *Lactobacillus* isolates during the fermentation of millet flour (Figure 8B). The general trend of the curves shows that the tryptophan content of the fermentation broth of *Lactobacillus pentosus S2, S3, S4, S5* and *S6* was decreasing at day 2 of fermentation and increasing at day 4*.* In contrast, the tryptophan content of *Lactobacillus pentosus S1* and *S7* increased first at day 2 of fermentation. The accumulation of tryptophan in the fermentation broth of millet flour was significantly higher than that in the fermentation broth of millet grains during 10 days of millet fermentation. The initial tryptophan content in the fermentation broth during the fermentation of millet flour was higher than that of millet grain fermentation, which indicates that the amino acids and other nutrients in millet entered the fermentation medium better after grinding and homogenization. The highest tryptophan content was found at day 8 of millet fermentation by *Lactobacillus pentosus S4*. Therefore, the millet flour was next fermented at different temperatures focusing on the effect of temperature on the tryptophan content of *Lactobacillus pentosus S4* fermented millet flour at the 8th day of fermentation, and the results proved that the highest tryptophan content was achieved at 30°C of fermentation temperature. The initial pH values of the seven strains during the fermentation of millet were 5.45, 5.42, 5.36, 5.21, 5.42, 5.48 and 5.53, respectively. The pH values dropped sharply during the first 2 days of fermentation, and almost ceased to change from the second day onwards. The final pH values of the fermentation broth were 4.97, 4.72, 4.58, 4.47, 4.72, 4.84, and 4.99, respectively (Figure 10).

#### 3.7.4 A strain of *Lactobacillus* pentosus with potential accumulation of tryptophan

*Lactobacillus pentosus S4* was isolated as a potential tryptophan accumulating isolate, with the highest tryptophan level of 238.43 mgL-1 in the fermentation broth, 1.80 times the original tryptophan content in the fermentation broth. The results showed that the growth of *Lactobacillus pentosus S4* was retarded and the highest biomass was lower than in MRS medium when millet grain was used as the fermentation medium, while the strain grew better when millet flour was used as the fermentation medium compared to millet grains. Further, *Lactobacillus pentosus S4* was fermented at 30°C for millet flour and millet grains, respectively, and the changes of L-tryptophan content in the fermentation broth were observed (Figure 8B).

The anabolic pathway of L-tryptophan is shown in Supplementary Figure S7, and the genes involved in L-tryptophan synthesis include, Group 1 (4.1.3.27): TRP3, TRP1, trpE, trpG, trpEG and trpGD; Group 2 (2.4.2.18): trpD and trpGD; Group 3 (5.3.1.24): TRP1, trpF, trpCF and priA; Group 4 (4.1.1.48): TRP3, TRP1, trpC and trpCF; Group 5 (4.2.1.20): trpA and trpB. The genes trpA (tryptophan synthase alpha), trpC and trpB were present in the *Lactobacillus pentosus SYBC M1* strain chain), trpB (tryptophan synthase beta chain), trpE (anthranilate synthase component I), trpG (anthranilate synthase component II), trpF (phosphoribosylanthranilate isomerase) and trpS (tryptophanyl-tRNA synthetase). *Lactobacillus pentosus S4* showed an overall increasing trend in tryptophan content when fermenting millet flour, and the tryptophan content was much higher than the other six isolates at the end of fermentation (day 10) accompanied by a decrease in phenylalanine and tyrosine content. This indicates that the pathway for the synthesis of tryptophan in the aromatic amino acid anabolic pathway in this bacterium is relatively smooth, probably due to full gene expression, high enzyme activity, or blockage of the branch pathways for the synthesis of the other two amino acids.

## 4 Discussion

This is the first report indicating the presence of acid-resistant lactic acid bacteria in the sour honey of Yunnan stingless bees in China. Data on the microbial community in Yunnan’s original sour honey are very limited; therefore, exploring the bacterial diversity in sour honey further is necessary. Here, the dominant genera of bacteria in sour honey collected from Yunnan, China, were *Carnimona*, *Clostridium*, *Lactobacillus*, *Bacillus,* and *Staphylococcus*. This knowledge improves the understanding of the community structure in sour honey bacteria at the genus level. The bacterial structures in the sour honey samples from four different producing areas were similar, but there were significant differences in the abundance levels of dominant bacteria, which might be related to the different flowers and plants available in different habitats. It has also been previously shown that sour honey is not conducive to the growth of certain microorganisms owing to its high acidity and high hydrogen peroxide (H_2_O_2_) contents (Ngalimat et al., 2019), but certain bacteria can still grow on the honey**.** In 2022, Flávia et al. found microorganisms, including *Escherichia coli*, yeast, and *Staphylococcus aureus*, as well as probiotics, such as *Bacillus*, in Brazilian honey samples from stingless bees (Izabely Nunes Moreira et al., 2023)**,** In the same year, Yaacob et al. isolated *Lactobacillus* and *Fructobacter* from fresh stingless honey from Malaysia (Syed Yaacob et al., 2018). In 2019, Zulkhairi Amin et al. isolated *Bacillus* from stingless honey collected in Malaysia (Zulkhairi Amin et al., 2020). Rosli et al. analyzed the bacterial diversity of local stingless bee honey in Malaysia in 2020 and found that the bacterial community was mainly composed of Firmicutes, among which the seven bacterial OTUs with the highest percentage of abundance were members of *Lactobacillus* (Rosli et al., 2020). This study isolated seven *lactobacillus* strains from sour honey. Among them, the strain *S4* (OM618128) showed potential acid-resistant abilities.

In this study, phylogenetic and comparative analyses of conserved regions of bacteria from four sour honey samples showed that the seven isolated strains were phylogenetically located in the *L. pentosus* subgroup. *Lactobacillus pentosus* has an extensive human nutrition history. It is widely present in a variety of fermented foods, including dairy products (Aureli et al., 2011), meat, and vegetable products (Aleklett et al., 2014), as well as in the human gastrointestinal tract (Olofsson et al., 2014), particularly in the mouth and gut. It facilitates digestion, aids in nutritional absorption, and controls pathogenic “harmful” microbes in the gastrointestinal system. In terms of genotype, the species *Lactobacillus plantarum, Lactobacillus pentosus*, and *Lactobacillus paraplantarum* are closely related and exhibit phenotypical traits that are strikingly similar. They are Facultative heterofermentative lactobacilli that are phylogenetically related by their 16S rRNA genes. Based on DNA-DNA hybridization data, Dellaglio et al. (Torriani et al., 2001b) established the genetic variability of the *L. plantarum group*. Three groups were categorized as *L. plantarum, L. pentosus* and *L. paraplantarum.* (Torriani et al., 2001a)**.** Genomic analysis also offers a comprehensive knowledge of the functional mechanisms underlying possible probiotic strains and their environmental adaptation, which would help further support the validity of their effectiveness.

In our study, potential acid-resistant *Lactobacillus* sp. *S4* (*Lactobacillus pentoses SYBC-M1*) was obtained after screening seven *Lactobacillus* isolates from sour honey. *Lactobacillus sp. S4* has an optimum growth temperature of 30°C and can survive at high salt concentrations. The lactic acid yield of *Lactobacillus sp. S4* was 17.62 g/L after a 24-h fermentation, the highest among the seven isolates. The physiological and biochemical properties indicated that *Lactobacillus sp*. S1, -5, and -6 survived normally when the salt concentration in the medium was 6%, indicating their resistance to some extent. *Lactobacillus sp*. S2 and -5 utilized D-triose, whereas only *Lactobacillus sp.* S4 utilized D-xylose and had a strong acid-production capacity. During the isolation of LAB, we used a common method of enriching the sour honey samples by culturing multiple rounds on an MRS liquid medium. This led to a significant increase in LAB content in the sour honey samples, making them the dominant species and allowing further isolation and identification. CaCO3 was added to MRS agar to separate the LAB. There is evidence that an MRS medium containing CaCO3 is the key to LAB isolation from sour honey. This is because sour honey characteristics, such as pH value, viscosity, and the inherent high concentration of sweeteners, are limiting factors for the accurate identification of LAB communities in ordinary MRS medium (Li et al., 2012).

Whole genome sequencing analysis was performed on *Lactobacillus pentosus* strain S4, a strain found to have potential acid resistance ability, degrade nitrite and accumulate tryptophan in millet fermentation applications; hence, this strain was named *Lactobacillus pentosus SYBC M1.* The genomic functional annotation of S4 (OM618128; *Lactobacillus pentosus SYBC-M1*) was annotated through the KEGG (Kyoto Encyclopedia of Genes and Genomes) GO, KEGG, NR, CAZy, CARD. Swiss-Prot and Pfam databases (blastp, evalue 1 105, identity 40%, and coverage 40%) were used to understand its genes’ biological roles further. Based on an average nucleotide identity (ANI) of 99.95%, genomic research revealed that the bacteria recovered from the stingless bee honey had the highest similarity to the type strain of *Lactobacillus pentosus*. The whole genome sequence was annotated for genes associated to carbohydrate metabolism (associated with prebiotic use), which are involved in adaptation of *L. pentosus SYB-MI* to the human gastrointestinal tract (GIT). Several genes, including glycoside hydrolases, glycoside transferases, and isomerases, as well as other enzymes involved in complex carbohydrate metabolism, particularly starch, raffinose, and levan, participate in coding for carbohydrate-modifying enzymes to modify oligo- and polysaccharides. Since these enzymes are involved in the metabolism and assimilation of complex carbohydrates that are not digested by human enzymes, they serve as key indicators of the bacteria’s adaptation to the GIT environment.

Genes involved in acquiring and using nutrients can be used to examine the strain’s capacity to survive in different environments. The antibiotic resistance analysis results show that 25 genes have been identified, with 19 genes for antibiotic resistance, 11 for antibiotic target, and 1 for antibiotic biosynthesis. A complete genomic sequence of a target microorganism should be studied to determine the phenotype and genotype of the strain before considering probiotics as a functional constituent. Knowing the genus and species of a probiotic strain is of the utmost importance since probiotic effects result from strain-specific probiotics. In addition, genome of targeted probiotic strain can be examined to determine if it contains genes that cause toxicity and antibiotic resistance (Mohammad et al., 2020) In addition, these genes can spread to other commensal microorganisms, providing important safety information. Potential probiotic bacteria do not have transferrable genes resistant to antibiotics for the evaluation of the safety aspects of human health (Mohan et al., 2017; Rodrigues et al., 2021). According to studies by Gueimonde et al. (2013), several *Lactobacillus* strains, including *L. casei* and *L. rhamnosus*, have an intrinsic resistance to vancomycin that cannot be passed on to other species or strains. However, the transfer risk for intrinsic resistance resulting from chromosomal mutation is also considered minimal in this case (Gueimonde et al., 2013). According to our research, *Lactobacillus pentosus SYBC- MI* is the strain that is resistant to vancomycin, kanamycin and streptomycin while susceptible or sensitive to erythromycin, chloramphenicol and ampicillin. Chloramphenicol susceptibility in *Lactobacillus* strains is widely known. On the other hand, some *Lactobacillus* strains, including *L. acidophilus, L. johnsonii,* and *L. reuteri,* have chloramphenicol resistance genes. It is important to remember that each prospective probiotic strain has unique characteristics for the antibiotic resistance. An important requirement for probiotics is the susceptibility of LAB strain to antibiotics. It is also debatable whether LAB strains typically considered safe should be sensitive or resistant to antibiotics.

According to (Araya et al., 2002), one of the essential conditions for a strain to adhere to the probiotic idea is that it must continue to be viable during GI transit. Probiotic bacteria must in reality, survive their passage through the stomach, accompanied by the strong acidity of the human gastric juice, to adhere to the intestinal mucosa, develop, and have therapeutic effects. In contrast, the pH ranges from 1 to 5 when fasting and rises to pH 3 and pH 5 when fed. The latter is mostly composed of pepsin and chlorhydric acid. During the screening of acid-resistant bacteria in our investigation, only one strain, S4, showed tolerance to pH 2.5 for 4 h. Using an acid and bile tolerance experiment, we evaluated the LAB isolates’ capacity to survive in an artificial gastric juice at pH 2.5, simulating conditions similar to those in the stomach. All strains have an appropriate level of salt tolerance. Four isolates (S1, S2, S4, and S6) showed highest bile resistance in the presence of 0.3% bile (above 50% compared to growth control). However, in the presence of 0.5% and 1% bile, S3 and S7 exhibited the highest survival rates (28% and 19%, respectively). Recent studies suggest 0.3% bile is the ideal concentration for evaluating bile-tolerant probiotic LABs (de Vries et al., 2006).

Acid resistance is considered a crucial factor in selecting possible probiotics since these severe conditions function as a natural barrier against living bacteria entering the intestinal tract (Aureli et al., 2011). The acidic environment in the stomach, where the pH is low (Huang et al., 2020), is the first obstacle to the host’s small intestine; nevertheless, ingested food has a buffering effect that typically raises the pH to 3; consequently, pH 3 is typically thought to be an ideal pH for a probiotic to survive (Olofsson et al., 2014). Additionally, bile salt is a harmful discharge from the small intestine. Lactobacilli use a variety of strategies to endure acid stress, including the maintenance of intracellular pH homeostasis, the repair of damaged proteins, and modifications to the cell membrane (Hill et al., 2018). A mechanism known as the acid tolerance response has also been shown to positively influence the acid resistance of several *Lactobacillus* strains when the cells have previously been exposed to moderately acidic conditions (Mesquita et al., 2017). The acid-resistant micro-ecological LAB preparations may be added to foods, or some micro-ecological preparations with acid-resistant LAB can be consumed directly. Recent studies (Fidanza et al., 2021) have provided some insight into the adaptive element of lactobacilli’s tolerance to acidic circumstances. However, there is still a lot of confusion about the molecular causes and processes of acid resistance.

Similarly, Pathogens mostly use hemolysis to enter the human body, which is the ability to lyse red blood cells by breaking the membrane (Wu et al., 2022). Therefore, it is important to determine whether the target strain being utilized as a probiotic has hemolytic activity. To assure probiotic safety, the FAO/WHO Working Group Guidelines for the Evaluation of Probiotics in Food recommend a hemolysis test (FAO/WHO, 2002). The zone produced by the tested bacteria can be classified as hemolysis (partial lysis exhibiting a deep green zone), hemolysis (the entire lysis indicating a clear zone), and hemolysis (no lysis resulting in no zone), depending on its characteristics. According to research on probiotics (Goldstein et al., 2015; Gomez-Perez et al., 2016), *Lactobacillus* spp. are known to exhibit both α-hemolytic activity as well as strong β-hemolytic activities. Due to the absence of a clear zone on the blood agar plate in this investigation, S4 showed γ-hemolysis. The whole-genome sequencing results, which demonstrated that this strain lacked any virulence genes (such as hemolysin), were consistent with this result. Testing the hemolytic activity of *L. pentosus SYBC- MI* in order to confirm its safety revealed that it should not have any hemolytic activities, particularly β-hemolysis, that is hazardous. Furthermore, even if probiotics are usually regarded as safe (GRAS; Won et al., 2021), it is advised to evaluate their hemolytic activity. In our investigation, *L. pentosus SYB-MI* showed -hemolysis, which is considered safe for use as a probiotic.

Probiotic bacteria survive and grow during fermentation and in the GIT. In order to eradicate pathogenic bacteria probiotics release antimicrobial metabolites such as organic acids, hydrogen peroxide, diacetyl, ethanol, phenols, and bacteriocin into their surrounding environment through a process known as competitive elimination (Stivala et al., 2021). According to other studies, the development of specific antibiotic resistance encourages the use of probiotics because they can be administered simultaneously with antibiotic therapy and facilitate the recovery of gut microbial balance. Probiotics must however, be safe for consumption by humans and must not have genes that confer antibiotic resistance (Shao et al., 2021). Before selecting the probiotic candidates that are qualified for the production of probiotic products, additional safety characteristics must be evaluated. In this study, we showed that the LABs produced from the fermented non-dairy product millet have antagonistic activity as well as probiotic potential (Heller, 2001).

All strains possess the properties required to function as probiotics. Additionally, they have proper hydrophobicity, aggregation, and adhesion qualities to adhere to the GIT. They establish colonies in the bowel and suppress harmful bacteria. Along with the previously indicated processes, they also showed inhibitory and eradicating properties against the biofilm produced by *P. aeruginosa* PTCC 1707, suggesting that they could be a viable option for dealing with bacterial biofilms. In order to expand our *in vitro* observations, it is essential to do some detailed *in vivo* investigations in addition to laboratory test models (*in vitro*). Coaggregation with foodborne pathogenic bacteria can be attributed to the inhibitory effects of the LAB strains (Bujnakova and Kmet, 2002). Autoaggregation of LAB is an important characteristic to measure probiotic potential since it is necessary for attachment to intestinal epithelial cells and inhibition of pathogen colonization in LAB strains (Puniya et al., 2016). There was significant autoaggregation (>50%) in *L. pentosus* (*SYBC-MI*) after 24 h of incubation, whereas the other strains showed trivial autoaggregation.

*In vitro* tests of *Lactobacillus pentosus SYB-MI*, a potential probiotic LAB isolated from stingless bee honey, showed that it has a number of probiotic properties, including the ability to auto-aggregate and co-aggregate with pathogenic bacteria, the ability to adhere to intestinal and vaginal cell lines, antagonistic activity against pathogens, and the fermentation of several prebiotics and lactose. However, the putative health-promoting abilities of this strain may depend on genetic traits and interactions within its ecological niche. For this reason, the whole-genome sequence and the subsequent annotation will increase our understanding of the functionality of this strain, its adaptation to the human gastrointestinal tract (GIT), the probiotic effect and its interaction within the host.

Similarly, Nitrites are potential carcinogens. Therefore, reducing nitrites in food is essential for food safety. Methemoglobinemia and acute poisoning are caused by ingesting significant amounts of nitrites. N-nitroso compounds are produced by the breakdown of nitrites and amines, which are the byproducts of protein and interact in suitable circumstances. Eighty percent of the over 100 N-nitroso compounds synthesized are potent animal carcinogens (Sallan et al., 2023). However, nitrites are frequently used in the meat industry to prevent the growth of *Clostridium botulinum* and as coloring agents. Therefore, reducing nitrites in food is highlighted in food safety research. The inoculation of *Lactobacillus* could prevent the accumulation of nitrites at high concentrations during the fermentation of different foods (Shao et al., 2021). In our research, the seven isolated strains were grown on MRS liquid medium that contained 125 g-mL^-1^ nitrite. *Lactobacillus* strain S4 shows the highest ability to degrade nitrite. The final pH of the culture medium was around 4-5, and the isolate exhibited a high ability to breakdown nitrite, exceeding 99.5%, according to the results of the 48-h incubation. Our isolated strain might be a potential probiotic by reducing nitrites in the food, increasing the gut’s non-immune antimicrobial defense. However, salivary nitrite can also combine with amines to create N nitroso compounds in stomach circumstances. The effectiveness of non-immune defense activity vs the potential development of N nitroso compounds in the stomach will determine how nitrate-reducing probiotics are used to treat intestinal diseases in the future.

During the fermentation of foxtail millet by seven *Lactobacillus pentosus* (S1–7) strains isolated from sour honey, a potential tryptophan accumulating isolate, *Lactobacillus pentosus SYBC-MI*, had reach a maximum tryptophan content of 238.43 mg L^-1^ in the fermentation broth, 1.80 times the initial tryptophan content in the fermentation broth. Millet fermentation with lactic acid bacteria, on the other hand, has a long history, and millet itself has a high potential for producing functional health foods. Carbohydrates, proteins, lipids, phytic acid, fiber, and other large molecules in it are broken down into smaller molecules that are more easily absorbed and utilized by humans during fermentation (Rodrigues et al., 2021). Traditional grains gain new flavor, nutritional value, or functions due to the new probiotic fermentation. The biomass and pH during fermentation were measured and analyzed; also, the free amino acid content during millet fermentation was measured. New nutritional values or new functions were applied to traditional grains through the fermentation of new probiotic bacteria.

In addition, a strain with the potential ability to accumulate tryptophan analyzed for the related genome. L-tryptophan is a sleep aid and a mental stabilizer, which can relieve depression. In millet-fermented foods, tryptophan content can be increased through fermentation, enhancing millet’s excellent therapeutic properties (Banwo et al., 2021; Ben-Miled et al., 2023). In the process of millet fermentation, it is important to use lactic acid bacteria that can produce acid quickly in a short period of time. This is because lactic acid bacteria with high acid-producing capacity can rapidly lower the pH of the fermentation broth, thereby inhibiting the growth of other microorganisms and resulting in improved fermentation performance. Tryptophan has a calming effect, and the sleep-aiding effect of millet may be related to its abundant tryptophan, but so far, very few studies have reported changes in tryptophan content during millet fermentation, and the paper measured the changes in tryptophan content in fermentation broth along with 17 common amino acids (Sutanto et al., 2022; Ben-Miled et al., 2023). Different taste-presenting amino acids make fermented foods rich in taste. For example, soy sauce’s freshness comes from the fresh amino acids and nucleotides produced by the microbial fermentation of proteins in raw materials. Soy sauce sweetness is also influenced to some extent by sweet amino acids and small-molecule peptides. The bitterness in soy sauce is not only related to magnesium sulfate contained in table salt, etc., but also to bitter amino acids. Taste-presenting amino acids need to be considered when fermenting millet (Stivala et al., 2021).

People are gradually adding LAB nutritional supplements with health functions to their diets (Siezen and van Hylckama Vlieg, 2011; Sauer et al., 2017). However, due to the stomach’s low-acid environment, some LAB strains have difficulty surviving. One of the essential conditions for a strain to adhere to the probiotic concept is that it must continue to be viable during gastrointestinal (GI) transit. Probiotic bacteria must survive the passage through the stomach, exposed to the high acidity of human gastric juice before they can stick to the intestinal mucosa, proliferate, and exert beneficial effects. It consists mainly of pepsin and chlorhydric acid. Acid resistance is considered an important factor in selecting potential probiotics since these harsh conditions work as a natural barrier against living bacteria entering the intestinal tract. Therefore, using acid-tolerance LAB in healthy foods and nutritional supplements may improve the effectiveness and ensure the quality of the products (Fidanza et al., 2021). The analysis of the bacterial composition in sour honey is extremely important to carry out further studies, and it is instructive for the development of probiotic products based on sour honey and secondary processing with sour honey, which will have a higher application and commercial value. The organic acid content in sour honey is higher than that of regular honey and has a lower pH, a quality that suggests the possible presence of lactic acid bacteria with better acid tolerance in sour honey; also, the high organic acid content may be due to the strong acid-producing lactic acid bacteria in it.

A concerted effort to identify and characterize LAB in these food sources is necessary and deserves scientific attention. Isolation of *lactobacillus* with probiotic potential and L-tryptophan accumulation evaluation is an interesting topic in research field. *Lactobacillus* species are known for their probiotic properties, which means they can provide health benefits to the host when consumed. L-tryptophan is an essential amino acid that plays a crucial role in various biological processes. It is important to note that the evaluation of L-tryptophan accumulation in *lactobacillus* strains may require optimization of growth conditions and media composition to maximize L-tryptophan production. Additionally, genetic engineering techniques can also be employed to enhance L-tryptophan production in *lactobacillus* strains if needed (Sutanto et al., 2022).

Overall, the isolation of *lactobacillus* with probiotic potential and the evaluation of L-tryptophan accumulation can be a fascinating research area with potential applications in the development of novel probiotic products with enhanced health benefits.

## 5 Limitations

Over the last few decades, there has been a remarkable record of articles on probiotics using *in vitro* and *in vivo* techniques, as well as their usage in clinical studies. Nonetheless, the development of a genomic approach to evaluate probiotic-related features is of interest. Overall, in addition to laboratory assessment models (*in vitro*), some thorough evaluations inside the human body (*in vivo*) are required to supplement our *in vitro* findings. Today, researchers are looking for a new way to controlling infectious diseases due to overuse of antibiotics and the growth of resistance microorganisms. To combat infectious diseases in humans and other animals, any effort to identify LAB is thus considered as an essential step toward the industrial production of these bacteria. The purpose of this study was to provide a more thorough investigation of the probiotic properties of LAB strains obtained from stingless bee honey through various *in vitro* tests to be used in the food industry, which would benefit both honeybees and humans through our diet by producing probiotic honey or other products.

## 6 Conclusion

In conclusion, this study investigated the presence of seven promising and novel strains of *Lactobacillus* with strong acid-resistant levels and acid-producing capacities isolated from the honey of the stingless bee in Yunnan, China. The results of a sequence alignment showed that all seven *Lactobacillus* strains were most similar to *L. pentoses*, with similarity scores of 99.47%, 99.79%, 99.89%, 99.26%, 99.47%, 99.47%, and 99.37%, respectively. The whole genome sequencing analysis for the specific *Lactobacillus sp*. named *Lactobacillus pentosus SYBC-MI,* which has potential applications in the food and feed industries. Furthermore, the results revealed the bacterial composition of sour honey in Yunnan, China, which increases our understanding of the dominant bacterial species in sour honey. The isolation of “good” strains of bacteria from sour honey, which have unexpected probiotic potential and human health benefits, presents a good reference value for developing related products in food and pharmaceuticals. According to the results of our studies, these LAB isolates provide a lot of potential for use in the future, as a source of probiotics for people, animals, and starting cultures for food applications. In the future, we can further explore the excellent lactic acid bacteria in sour honey and investigate the key taxa and their key roles in the environment. The functional genes of the different metabolic pathways of *Lactobacillus pentosus* and *Lactobacillus plantarum* could be considered for further study and exploration in the future. The genomes of the isolated *Lactobacillus* strains can be further explored for better applications. This will increase the knowledge of sour honey in Yunnan, China, and make it a new source of excellent strains.

## Data Availability

The datasets presented in this study can be found in online repositories. The names of the repository/repositories and accession number(s) can be found in the article/Supplementary Material.
