# Supplementary figures and images for: Bacterial diversity of stingless bee honey in Yunnan, China: isolation and genome sequencing of a novel acid-resistant Lactobacillus pentosus (SYBC-MI) with probiotic and L. tryptophan producing potential via millet fermentation
Source: Front Bioeng Biotechnol. 2023 Dec 1;11:1272308. doi: 10.3389/fbioe.2023.1272308 (PMC10722240; doi:10.3389/fbioe.2023.1272308)

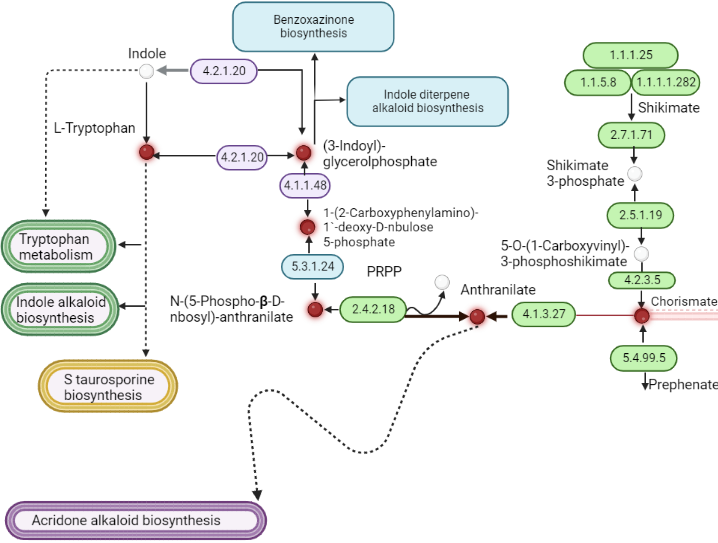

Supplement: Supplementary file 1 [file Image6.TIF]

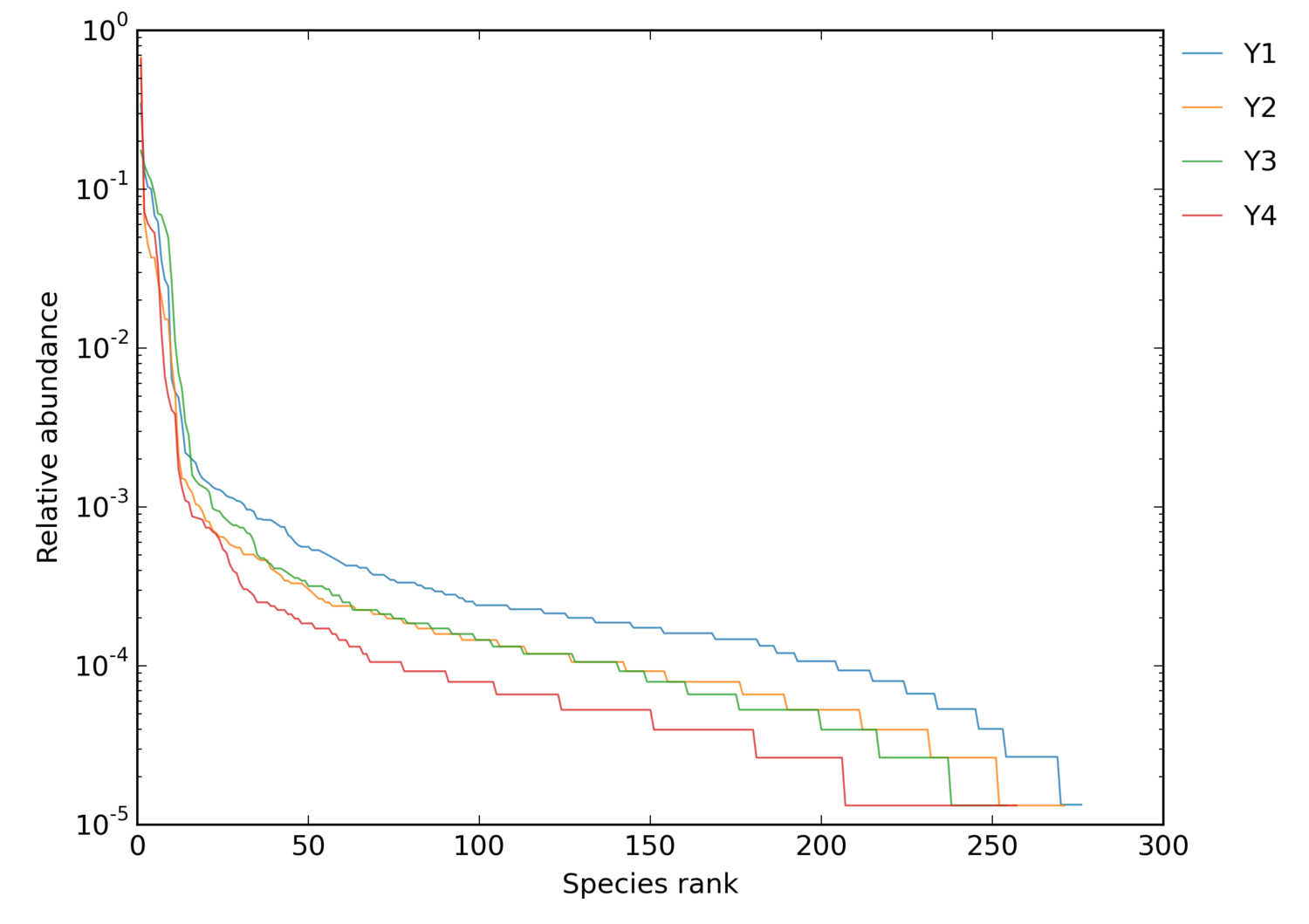

Supplement: Supplementary file 3 [file Image3.TIF]

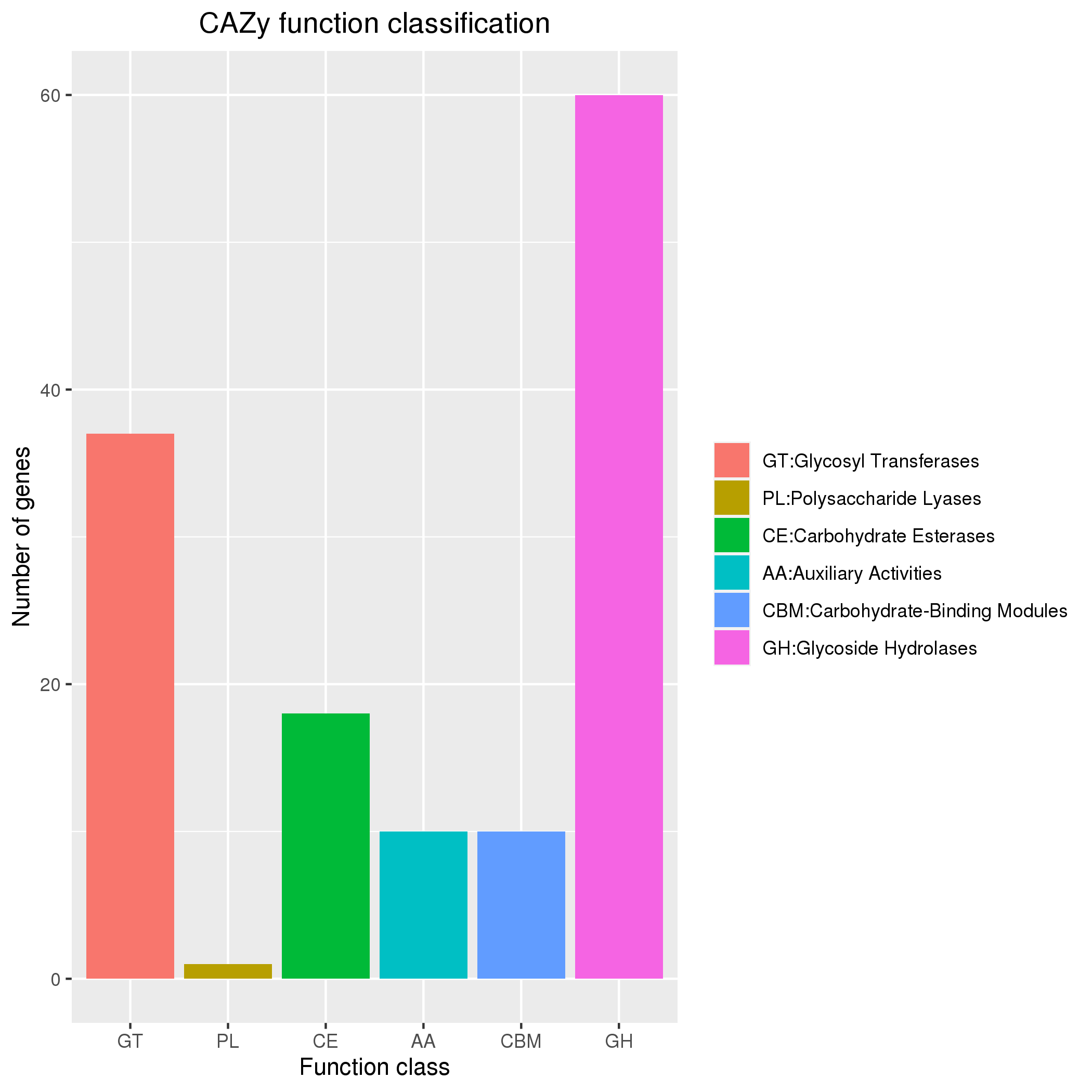

Supplement: Supplementary file 4 [file Image4.TIF]

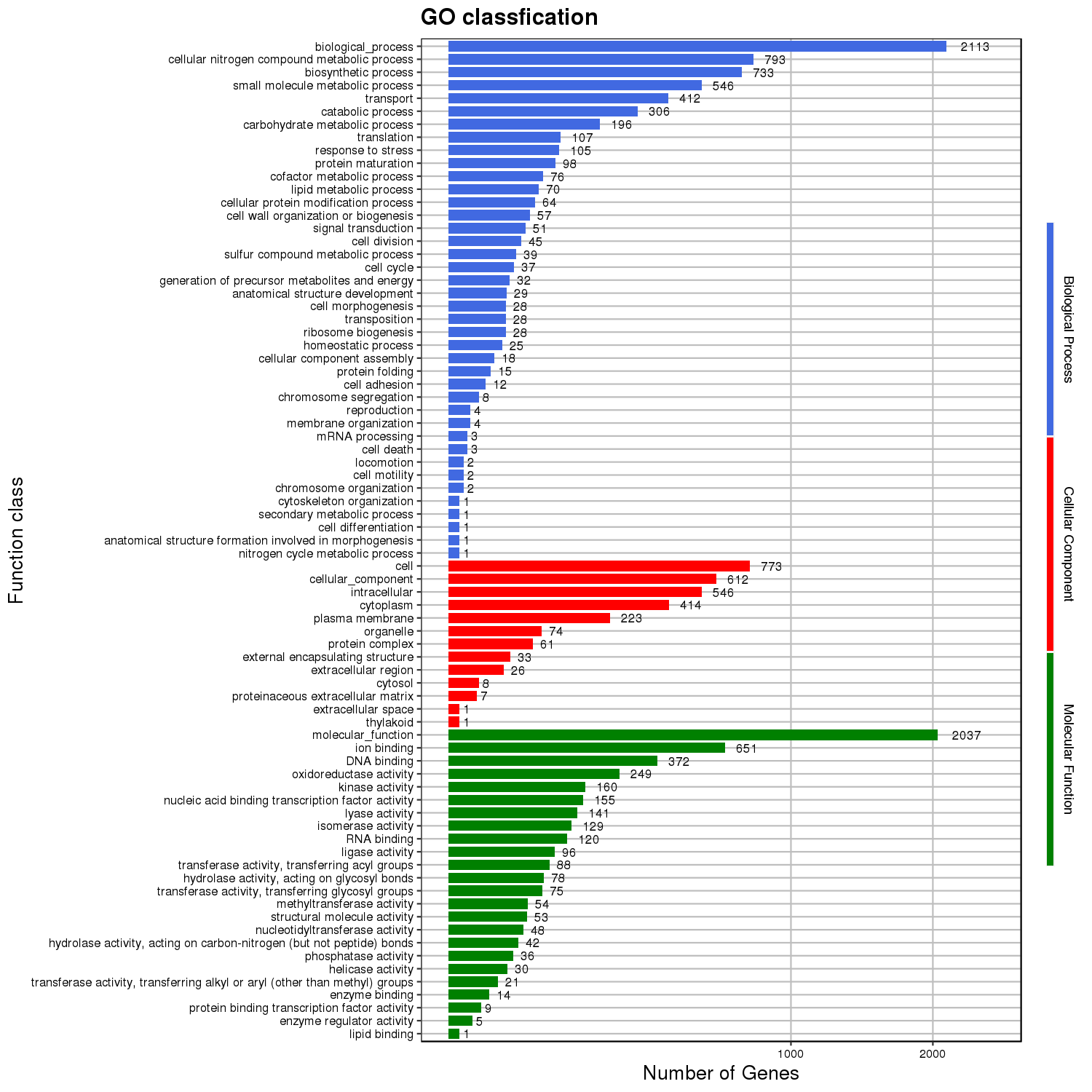

Supplement: Supplementary file 5 [file Image2.TIF]

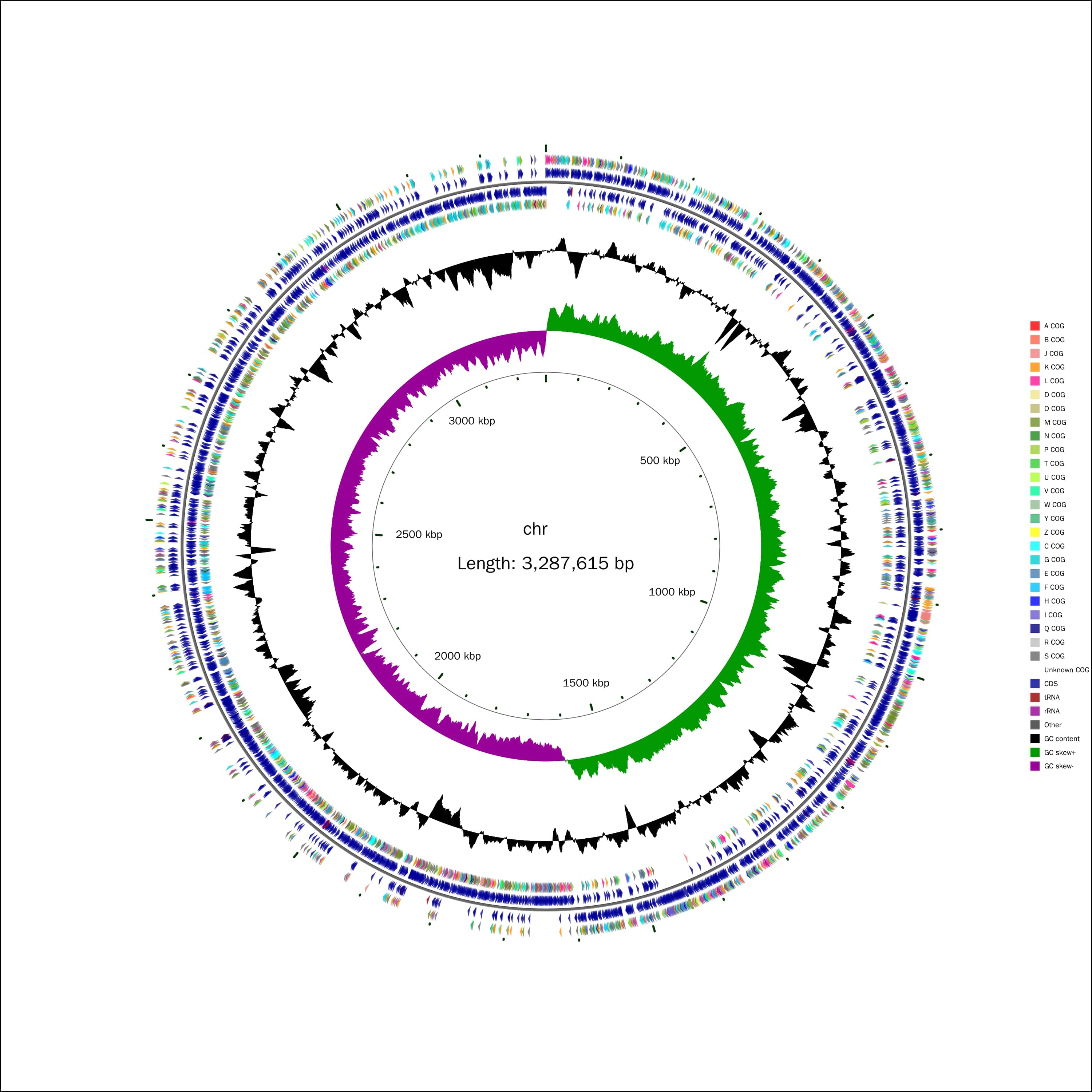

Supplement: Supplementary file 6 [file Image7.JPEG]

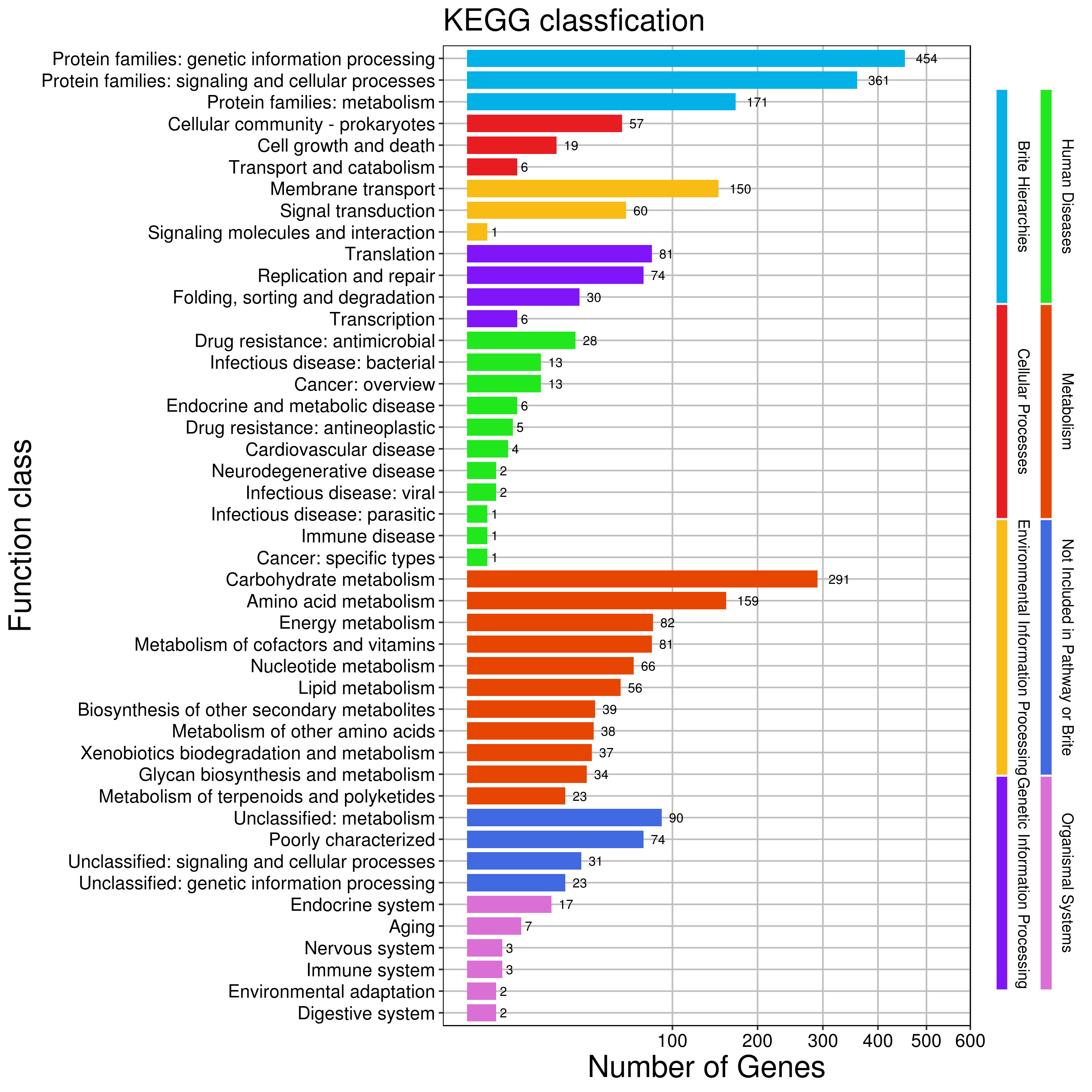

Supplement: Supplementary file 7 [file Image1.TIF]

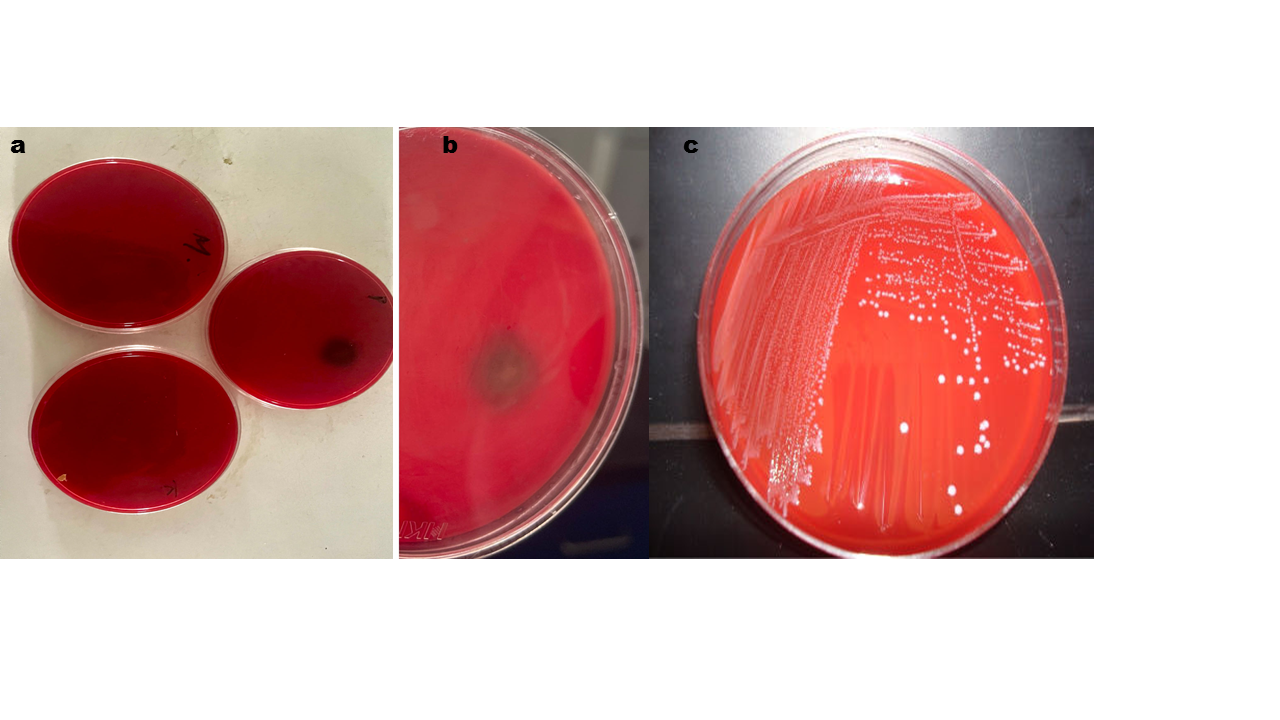

Supplement: Supplementary file 8 [file Image5.TIF]
