# Supplementary material for: Bacterial diversity of stingless bee honey in Yunnan, China: isolation and genome sequencing of a novel acid-resistant Lactobacillus pentosus (SYBC-MI) with probiotic and L. tryptophan producing potential via millet fermentation
Source: Front Bioeng Biotechnol. 2023 Dec 1;11:1272308. doi: 10.3389/fbioe.2023.1272308 (PMC10722240; doi:10.3389/fbioe.2023.1272308)
Supplement: Supplementary file 2 [file Table1.docx]

**Supplementary Tables:**

Table.S1. Functional gene annotation of *L. pentosus SYBC-M1.*

| Type | No. Of Genes | Percentage % |
| --- | --- | --- |
| NR | 3,124 | 99.8402 |
| eggNOG | 2,641 | 84.404 |
| KEGG | 1,431 | 45.7335 |
| Swiss-prot | 2,131 | 68.1048 |
| GO | 2,224 | 71.077 |
| Secretory proteins | 76 | 2.43% |
| CARD | 25 | Above 45% |
|  |  |  |

Table S2. Prophage prediction in the genome

| Prophage ID | Start | Stop | Start of attL | End of attL | start of AttR | End of AttR | |
| --- | --- | --- | --- | --- | --- | --- | --- |
| pp1 | 557,639 | 604,567 | 555,760 | 555,771 | 601,776 | 601,787 |  |
| pp2 | 910,152 | 925,913 | 909,022 | 909,036 | 945,352 | 945,366 |  |
| pp3 | 1,399,726 | 1,408,463 | 1,398,596 | 1,398,610 | 1,434,926 | 1,434,940 |  |
| pp4 | 1,453,464 | 1,471,559 | 1,452,334 | 1,452,348 | 1,488,664 | 1,488,678 |  |
| pp5 | 1,801,916 | 1,850,570 | 1,801,834 | 1,801,847 | 1,849,356 | 1,849,369 |  |
| pp6 | 2,150,823 | 2,235,467 | 2,152,358 | 2,152,370 | 2,236,682 | 2,236,694 |  |

Table S3. Selected results of secretory protein prediction

| ORF name | Cmax | Pos | Ymax | Pos | Smax | Pos | Smean | D |  |
| --- | --- | --- | --- | --- | --- | --- | --- | --- | --- |
| chr_88 | 0.272 | 33 | 0.441 | 29 | 0.826 | 25 | 0.608 | 0.506 |  |
| chr_129 | 0.81 | 31 | 0.844 | 31 | 0.985 | 17 | 0.931 | 0.878 |  |
| chr_156 | 0.192 | 32 | 0.354 | 29 | 0.768 | 3 | 0.694 | 0.487 |  |
| chr_164 | 0.216 | 31 | 0.366 | 31 | 0.747 | 24 | 0.584 | 0.451 |  |
| chr_176 | 0.713 | 29 | 0.795 | 29 | 0.968 | 27 | 0.839 | 0.812 |  |
| chr_179 | 0.213 | 31 | 0.398 | 22 | 0.937 | 20 | 0.864 | 0.58 |  |
| chr_180 | 0.292 | 39 | 0.412 | 39 | 0.869 | 26 | 0.707 | 0.527 |  |
| chr_240 | 0.701 | 28 | 0.82 | 28 | 0.985 | 19 | 0.959 | 0.874 |  |
| chr_263 | 0.613 | 27 | 0.718 | 27 | 0.954 | 8 | 0.878 | 0.78 |  |
| chr_30 | 0.455 | 29 | 0.541 | 29 | 0.799 | 2 | 0.679 | 0.595 |  |
|  |  |  |  |  |  |  |  |  |  |

Table S4.The antibiotic resistance analysis

| Seq ID | Property | Number of Genes | Percentage (%) | |  |
| --- | --- | --- | --- | --- | --- |
| chr | Antibiotic Resistance | 19 | 0.607 |  |  |
| chr | Antibiotic Target | 11 | 0.352 |  |  |
| chr | Antibiotic Biosynthesis | 1 | 0.032 |  |  |
| chr | Total genes | 25 | 0.799 |  |  |
| plasmid1 | Antibiotic Resistance | 0 | 0 |  |  |
| plasmid1 | Antibiotic Target | 0 | 0 |  |  |
| plasmid1 | Antibiotic Biosynthesis | 0 | 0 |  |  |
| plasmid1 | Total genes | 0 | 0 |  |  |

Table S5. Biomass of millet fermented by Lactobacillus

| Fermentation time  （d） | Biomass of each sour honey lactic acid bacteria in fermentation broth (CFU-mL-1)（CFU·mL^-1^） | | | | | | |
| --- | --- | --- | --- | --- | --- | --- | --- |
|  | S1 | S2 | S3 | S4 | S5 | S6 | S7 |
| 0 | 6.8×10^7^ | 6.2×10^7^ | 3.7×10^7^ | 5.2×10^7^ | 5.8×10^7^ | 6.0×10^7^ | 7.2×10^7^ |
| 2 | 6.0×10^8^ | 6.3×10^8^ | 1.3×10^8^ | 6.3×10^8^ | 6.7×10^8^ | 4.3×10^8^ | 7.3×10^8^ |
| 4 | 8×10^8^ | 1.8×10^8^ | 3.0×10^7^ | 1.8×10^8^ | 4.0×10^8^ | 3.8×10^8^ | 1.8×10^8^ |
| 6 | 6.2×10^7^ | 1.5×10^7^ | 1.6×10^7^ | 5.1×10^7^ | 1.0×10^8^ | 3.6×10^7^ | 9.0×10^7^ |
| 8 | 3.3×10^7^ | 4.3×10^7^ | 7.0×10^6^ | 4.3×10^7^ | 4.3×10^7^ | 3.4×10^7^ | 8.6×10^7^ |
| 10 | 3.0×10^7^ | 8.9×10^6^ | 8.9×10^6^ | 3.6×10^7^ | 2.1×10^7^ | 1.5×10^7^ | 1.2×10^7^ |

Table S6. Concentration of free amino acids in millet fermentation broth at the beginning and end of fermentation (except tryptophan)

| Free amino acid initial fermentation | Day 0-1 | Fermentation broth of each strain on day 10 of fermentation (mg-L-^1^)（mg·L^-1^） | | | | | | |
| --- | --- | --- | --- | --- | --- | --- | --- | --- |
|  |  | S1 | S2 | S3 | S4 | S5 | S6 | S7 |
| Total content | 983.53  ±54.40 | 893.92  ±69.17 | 977.96  ±7.57 | 812.98  ±20.41 | 784.89  ±32.08 | 795.68  ±5.44 | 800.36  ±24.31 | 943.83  ±73.75 |
| Essential amino acids： |  | | | | | | | |
| Therionine | 17.72  ±1.77 | 37.21  ±10.29 | 35.62  ±5.84 | 63.76  ±6.24 | 49.67  ±26.99 | 84.54  ±15.41 | 65.64  ±9.59 | 16.50  ±0.34 |
| Valine | 13.79  ±8.30 | 2.29  ±0.84 | 26.86  ±0.81 | 1.63  ±0.12 | 23.01  ±3.35 | 23.64  ±4.03 | 5.51  ±2.65 | 36.21  ±4.28 |
| Isoleucine | 14.24  ±4.22 | 3.73  ±1.99 | 28.56  ±2.14 | 5.46  ±5.07 | 44.91  ±3.70 | 30.49  ±3.21 | 12.33  ±4.70 | 6.49  ±0.19 |
| Leucine | 17.51  ±7.43 | 5.30  ±3.74 | 60.68  ±1.84 | 4.02  ±0.55 | 13.26  ±5.25 | 24.00  ±5.97 | 22.11  ±7.91 | 4.69  ±1.27 |
|  | 31.34  ±21.95 | 9.13  ±2.83 | 98.30  ±22.03 | 12.13  ±4.02 | 17.03  ±3.47 | 12.93  ±3.72 | 6.46  ±4.24 | 24.98  ±2.39 |
| Phenylalanine | 24.07  ±11.26 | 10.28  ±0.84 | 18.06  ±2.96 | 2.38  ±0.36 | 23.92  ±6.78 | 6.88  ±5.19 | 7.32  ±3.49 | 8.79  ±3.82 |
| Lysine | 18.36  ±7.58 | 11.34  ±2.74 | 25.33  ±2.74 | 12.19  ±2.17 | 14.97  ±1.40 | 12.79  ±5.12 | 10.17  ±1.82 | 31.56  ±5.78 |

Note: Data are expressed as the mean ± standard deviation of three measurements.
